# Multifaceted role of TREX2 in the skin defense against UV-induced skin carcinogenesis

**DOI:** 10.18632/oncotarget.4296

**Published:** 2015-06-15

**Authors:** Joan Manils, Diana Gómez, Mercè Salla-Martret, Heinz Fischer, Jason M. Fye, Elena Marzo, Laura Marruecos, Inma Serrano, Rocío Salgado, Juan P. Rodrigo, Juana M. Garcia-Pedrero, Anna M. Serafin, Xavier Cañas, Carmen Benito, Agustí Toll, Sònia-Vanina Forcales, Fred W. Perrino, Leopold Eckhart, Concepció Soler

**Affiliations:** ^1^ Departament de Patologia i Terapèutica Experimental, Facultat de Medicina, Campus de Bellvitge, Universitat de Barcelona, L'Hospitalet de Llobregat, Barcelona, Spain; ^2^ Research Division of Biology and Pathobiology of the Skin, Department of Dermatology, Medical University of Vienna, Vienna, Austria; ^3^ Department of Biochemistry, Wake Forest School of Medicine, Winston-Salem, North Carolina, USA; ^4^ Dermatology Departament, Hospital del Mar, Barcelona, Spain; ^5^ Department of Otolaryngology, Hospital Universitario Central de Asturias, Instituto Universitario de Oncología del Principado de Asturias (IUOPA), Universidad de Oviedo, Oviedo, Spain; ^6^ Plataforma de Recerca Aplicada en Animal de Laboratori, Parc Científic de Barcelona, Barcelona, Spain; ^7^ Protecció Radiològica, Universitat de Barcelona, Barcelona, Spain; ^8^ Institute of Predictive and Personalized Medicine of Cancer, Badalona, Barcelona, Spain

**Keywords:** TREX2, skin carcinogenesis, UV radiation, DNA damage, inflammation

## Abstract

TREX2 is a 3′-DNA exonuclease specifically expressed in keratinocytes. Here, we investigated the relevance and mechanisms of TREX2 in ultraviolet (UV)-induced skin carcinogenesis. TREX2 expression was up-regulated by chronic UV exposure whereas it was de-regulated or lost in human squamous cell carcinomas (SCCs). Moreover, we identified SNPs in the *TREX2* gene that were more frequent in patients with head and neck SCCs than in healthy individuals. In mice, TREX2 deficiency led to enhanced susceptibility to UVB-induced skin carcinogenesis which was preceded by aberrant DNA damage removal and degradation as well as reduced inflammation. Specifically, TREX2 loss diminished the up-regulation of IL12 and IFNγ, key cytokines related to DNA repair and antitumor immunity. In UV-treated keratinocytes, TREX2 promoted DNA repair and passage to late apoptotic stages. Notably, TREX2 was recruited to low-density nuclear chromatin and micronuclei, where it interacted with phosphorylated H2AX histone, which is a critical player in both DNA repair and cell death. Altogether, our data provide new insights in the molecular mechanisms of TREX2 activity and establish cell autonomous and non-cell autonomous functions of TREX2 in the UVB-induced skin response.

## INTRODUCTION

TREX2 is a non-processive 3′–5′ exonuclease that may be involved in genome maintenance by editing 3′-ends in multiple pathways, ranging from DNA replication, recombination and repair to degradation. The structure, biochemical properties, and molecular mechanisms of TREX2 in DNA interactions and catalysis *in vitro* have been well characterized [1–3], but less is known about its functions and mechanisms *in vivo*. Although studies in embryonic stem cells have shown that *Trex2* deletion leads to spontaneous chromosomal rearrangements [4] and inactivation of *Trex2* exonuclease leads to double strand breaks (DSBs) [5] *Trex2* knockout mice (*Trex2*^−/−^) do not exhibit a spontaneous cancer-prone phenotype or chromosomal instability [6], as would be expected if TREX2 had a relevant DNA-editing role. However, a chemically-induced skin carcinogenesis protocol revealed impaired keratinocyte apoptosis and an increased tumor incidence in the absence of TREX2 [6]. Furthermore, TREX2 ectopic co-expression with rare-cleaving endonucleases demonstrates that TREX2 can alter the normal function of end-joining DSB repair pathways. TREX2 processing of 3′-ends abrogates precise rejoining of endonuclease-induced breaks through the non-homologous end joining (NHEJ) pathway that determines resolution of mutagenic breaks [7–9]. Recent work in a heterologous cellular context shows that TREX2 can be a component of the error-free post-replication repair (EF-PRR) system, which is required to bypass damage that blocks replicative DNA polymerases [10]. Thus, upon DNA damage TREX2 can potentially participate in multiple pathways related to maintenance of genome stability. However, the physiological relevance of TREX2 in native cells remains elusive.

Unlike the ubiquitous expression of other 3′ exonucleases, TREX2 is specifically expressed in keratinocytes [6], cells that are naturally exposed to exogenous DNA damaging effects, such as UVB radiation and environmental chemicals, which are major risk factors for the development of cutaneous squamous cell carcinomas (cSCCs) and head and neck SCCs (HNSCCs), respectively [11, 12]. Keratinocytes display several efficient protective mechanisms to counteract harmful effects of UVB on DNA, most likely as result of evolutionary pressure. In response to UVB, keratinocytes undergo either apoptosis or cell cycle arrest depending on their intracellular status, the extent and persistence of DNA damage, and the balance between survival and death signals [13, 14]. In addition, UVB exposure triggers a coordinated immune response to maintain skin homeostasis [15, 16]. Deregulation of any of these pathways may contribute to the onset of UVB-induced skin cancers.

In the present study, we demonstrate a multifunctional role for TREX2 in the skin response to UVB using *Trex2*^−/−^ mice and keratinocytes. Upon UVB exposure, TREX2 promotes the elimination of DNA damage through DNA repair and cell death mechanisms, and drives an anti-tumoral immunity, thus suppressing skin carcinogenesis. Mechanistically, we provide evidences that TREX2 interacts with γH2AX. Furthermore, consistent with a role for TREX2 in keratinocyte-derived carcinogenesis, sequence polymorphisms in the *TREX2* gene and aberrant TREX2 expression were found in human squamous carcinogenesis.

## RESULTS

### TREX2 is altered in UVB-irradiated skin, actinic keratosis lesions and squamous cell carcinomas

**Figure 1:**
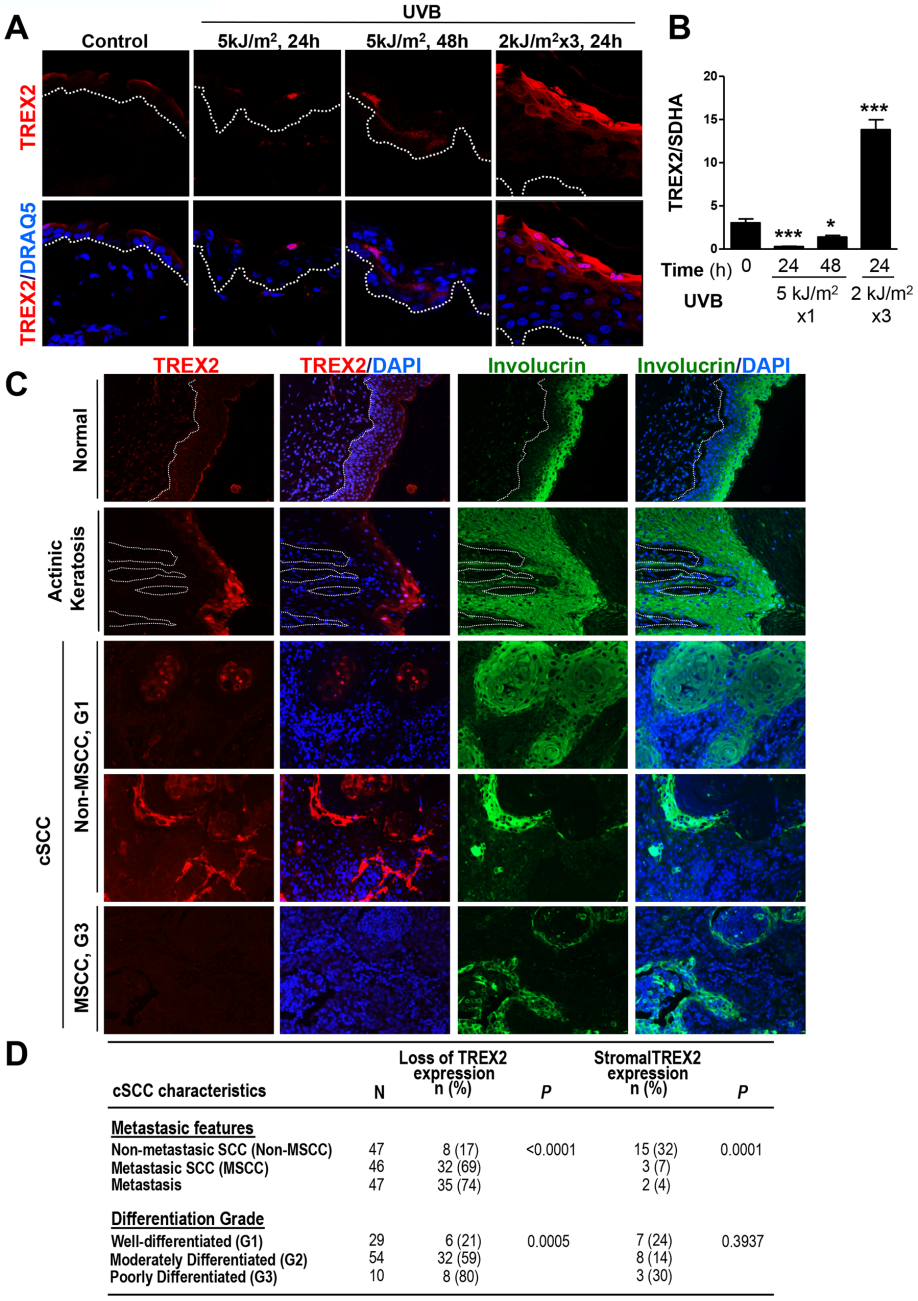
TREX2 expression is up-regulated in chronic UVB-irradiated skin and actinic keratosis lesions, whereas it is deregulated or lost in cSCC **A.** TREX2 immunostaining and **B.** mRNA expression in acute and chronic UVB-irradiated mouse skin. Graph shows mean and SEM of at least 5 mice. Differences between untreated and UVB-treated skin: unpaired Student's *t* test (*P < 0.05; ***P < 0.001). **C.** TREX2 expression in healthy human skin, actinic keratosis lesions and cSCCs determined by immunofluorescence. Nuclei were counterstained with DRAQ5 or DAPI, as indicated. White dotted lines demarcate the epidermal-dermal border. Original magnification, 40 × (A) and 20 × (C) **D.** Evaluation of TREX2 expression in cSCCs for features of metastasis and differentiation. *P* values of Chi-square test are indicated.

We first analyzed whether the UVB radiation targets TREX2 expression on mouse skin. Notably, acute UVB irradiation transiently down-regulated, while chronic exposure highly up-regulated TREX2 expression, as clearly showed by immunofluorescence (Fig. 1A) and RT-qPCR (Fig. 1B) assays. Immunofluorescence images also reveal that TREX2 expression was mainly detected in the suprabasal layers of the epidermis, where keratinocytes became differentiated as evidenced by keratin 10 (K10) expression (Supplemental Fig. 1A). Consistently, in primary keratinocyte cultures, strong TREX2 staining was associated with the expression of involucrin (Supplemental Fig. 1B). Both K10 and involucrin expression are induced during keratinocyte differentiation [17]. The specificity of TREX2 immunolabeling was validated by the absence of staining in the skin and keratinocytes from *Trex2*^−/−^ mice [6] (Supplemental Fig. 1A-1B). Therefore, these results demonstrated that TREX2 expression in keratinocytes was regulated during differentiation and by UVB radiation. The analysis of TREX2 by immunofluorescence in human precancerous actinic keratosis lesions and keratinocyte-derived tumors, including cSCCs (Fig. 1C-1D) and HNSCCs (Table 1), revealed a frequent deregulated expression of this exonuclease in squamous carcinogenesis. TREX2 staining in healthy skin was primarily detected in the granular cell layer of the epidermis. Interestingly, in actinic keratosis lesions, TREX2 expression increased in upper keratinocyte layers and displayed predominantly nuclear localization. Furthermore, TREX2 levels and patterns of expression in cSCCs varied depending on the degree of tumor differentiation and metastasis. Thus, TREX2 staining exhibited a heterogeneous pattern throughout the tumor, but was relatively intense in differentiated areas of mainly non-metastatic and well-differentiated tumors. In addition, strong TREX2 staining was also detected in tumor-associated stroma for a significant subset of non-metastatic cSCCs. In contrast to this, loss of TREX2 expression was significantly associated to metastatic cSCC, and poorly differentiated tumors. Analogously, in HNSCCs, the absence of TREX2 expression was significantly associated with nodal metastasis and advanced disease stages (Table 1). Curiously, in cSCCs the percentage of non-metastasic tumors with loss of TREX2 expression is lower (17%) than in HNSCC (59%).

**Table 1:** Relationship between TREX2 expression and clinicopathological features of HNSCC patients

HNSCC Characteristic	*N*	Loss of TREX2 expression *n* (%)	*P*	Stromal TREX2 expression n (%)	*P*
**pT classification**					
T1-T2	41	28 (68)	0.719^#^	12 (29)	0.251^#^
T3	47	35 (74)		7 (15)	
T4	45	34 (76)		9 (20)	
**pN classification**					
N0	44	26 (59)	0.014^&^	14 (32)	0.042^&^
N1-3	89	71 (80)		14 (16)	
**Disease stage**					
I-II	19	10 (53)	0.048^&^	8 (42)	0.029^&^
III-IV	114	87 (76)		20 (18)	
**Pathological grade**					
Well-differentiated	38	28 (74)	0.323^#^	8 (21)	0.207^#^
Moderately Differentiated	59	46 (78)		9 (15)	
Poorly Differentiated	36	23 (64)		11 (31)	
**Site**					
Pharynx	65	51 (78)	0.177^&^	12 (18)	0.528^&^
Larynx	68	46 (68)		16 (24)	

Number and percentage of tumors showing either a loss of TREX2 expression in the squamous epithelia or gain of TREX2 expression in the tumor-associated stroma, with statistical evaluation.

# Chi-square and

& Fisher's exact tests.

**Figure 2:**
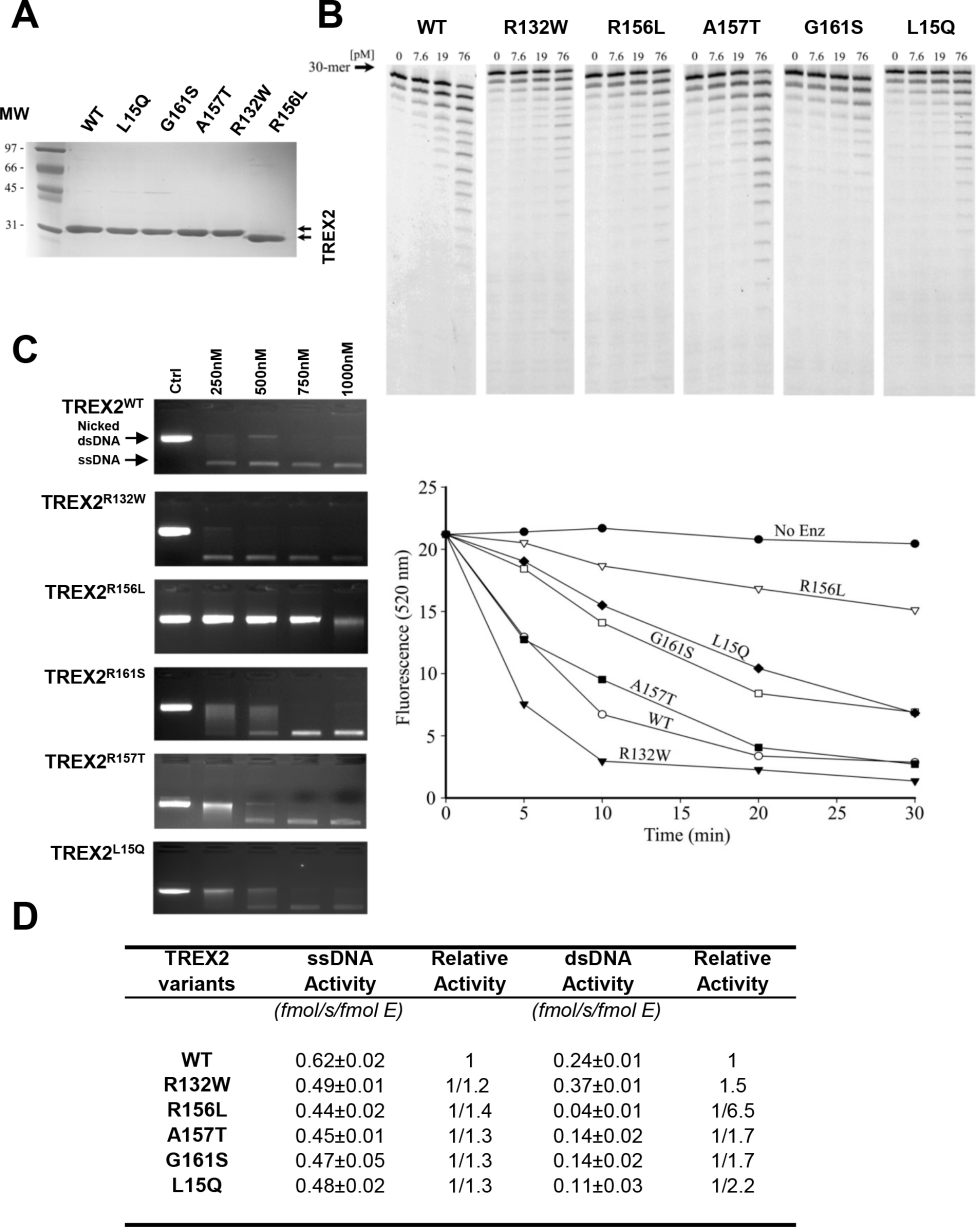
Exonuclease activities of wt and mutant TREX2 variants **A.** SDS-PAGE analysis of the purified TREX2 variants. The migration positions of the molecular weight (MW) standards are indicated. The size difference observed in the R156L variant is due to the plasmid construct used for its cloning and expression, which left six fewer residues at its N-terminus compared to the constructs used for the other variants. **B.** ssDNA exonuclease activities. **C.** dsDNA exonuclease activities. Exonuclease titration reactions and time course reactions are shown. The positions of migration of Form II nicked dsDNA (*Nicked dsDNA*) and circular ssDNA (*ssDNA*) are indicated. **D.** Relative ssDNA and dsDNA exonuclease activities.

We next compared genetic variability of the exonuclease TREX2 by sequencing the human *TREX2* gene in tumor and blood samples obtained from HNSCC patients and healthy controls. Five rare non-synonymous SNPs (L15Q, R132W, R156L, A157T and G161S) and two synonymous SNPs were identified in the *TREX2* coding sequence (Table 2). In addition, one common and nine rare SNPs were detected in the non-coding intron sequence upstream of the *TREX2* coding region. SNPs were present in both blood and tumor samples indicating a germline origin. These single amino acid variants were expressed in homozygosis because the *TREX2* gene is located on the X chromosome and all coding variants were found in male patients. The relatively robust activities of all TREX2 variants using ss- and dsDNA assays demonstrate that these variants are not complete loss-of-function mutations (Fig. 2). Overall, the relatively modest 2-fold differences in ssDNA and dsDNA degradation activities detected in the R132W, A157T, G161S and L15Q variants seem unlikely to affect overall cellular exonuclease activity, in contrast to the 6.5-fold lower dsDNA degradation activity detected in the R156L variant. However, we cannot rule out a greater level of TREX2 exonuclease dysfunction caused by these variants within the context of chromatin DNA degradation in the cell. Also, functional consequences of these genetic variants might be mediated through altered *in vivo* protein-protein or protein-DNA interactions. Consistent with this idea the R132, R156, A157, and G161 residues are positioned on the TREX2 surface ideally located to interact with other cellular molecules or with DNA [3]. The L15 residue interacts hydrophobically with L126 and L127 to appropriately position the alpha helix on which the R132 resides. The R156, A157, and G161 residues are positioned on the same flexible loop between helices α6–α7 containing R163, R165, and R167 that we previously demonstrated directly contribute to DNA binding in the TREX2 enzyme [3]. Thus, the identified TREX2 variants are in positions to disrupt interactions by multiple mechanisms. Interestingly, when grouping all rare *TREX2* SNPs, the frequency in affected individuals was higher than in controls (Table 2).

**Table 2:** Genetic variations and frequencies of the human *TREX2* gene in patients with HNSCC and in healthy individuals

Position on X Chromosome	Accession number	Major allele	Minor allele	Position vs first ORF nucleotide	Amino acid	Cases (*n* = 190)		Controls (*n* = 189)	
						Minor allele samples *n*	Frequency (%)	Minor allele samples *n*	Frequency (%)
152712935	rs34107439	C	A	(−)2047		3	1.579	2	1.058
152712811	rs35426191	G	A	(−)1922		1	0.526	0	0.000
152712525	rs371785311	G	-	(−)1637		5	2.632	0	0.000
152712460	rs3020962	T	G	(−)1571		53	27.895	54	28.571
152712310	rs191272965	G	A	(−)1421		1	0.526	0	0.000
152712310	rs191272965	G	T	(−)1421		1	0.526	1	0.529
152712115	rs568161256	G	A	(−)1227		1	0.526	0	0.000
152711255	rs533718948	T	C	(−)367		1	0.526	0	0.000
152711060	rs138397873	T	A	(−)172		2	1.053	0	0.000
152710970	rs62594087	G	A	(−)82		1	0.526	2	1.058
152710845	rs201756523	A	T	(+)44	**L15Q**	1	0.526	0	0.000
152710495	rs547615095	G	A	(+)274	**R132W**	1	0.526	1	0.529
152710422	rs199587066	C	A	(+)467	**R156L**	2	1.053	0	0.000
152710420	rs570718370	C	T	(+)469	**A157T**	1	0.526	0	0.000
152710408	rs200659060	C	T	(+)481	**G161S**	2	1.053	2	1.058
152710373	rs150316545	G	A	(+)516	**L172L**	0	0.000	1	0.529
152710236	ss1026802600	A	G	(+)651	**R217R**	6	3.158	2	1.058

	Number in affected individuals (Frequency)	Number in control individuals (Frequency)	*P* value
**Nonsynonymous SNPs**	7 /190 ( 0.037)	3/189 (0.016)	0.337
**Minor SNPs**	29/190 (0.153)	11/189 (0.058)	0.004
**Common SNPs**	53/190 (0.279)	54/189 (0.286)	0.910
**Minor & Common SNPs**	82/190 (0.431)	65/189 (0.344)	0.114

*P* values from Fisher's exact test are indicated.

### TREX2 deficiency enhances susceptibility to UVB-induced skin tumorigenesis

**Figure 3:**
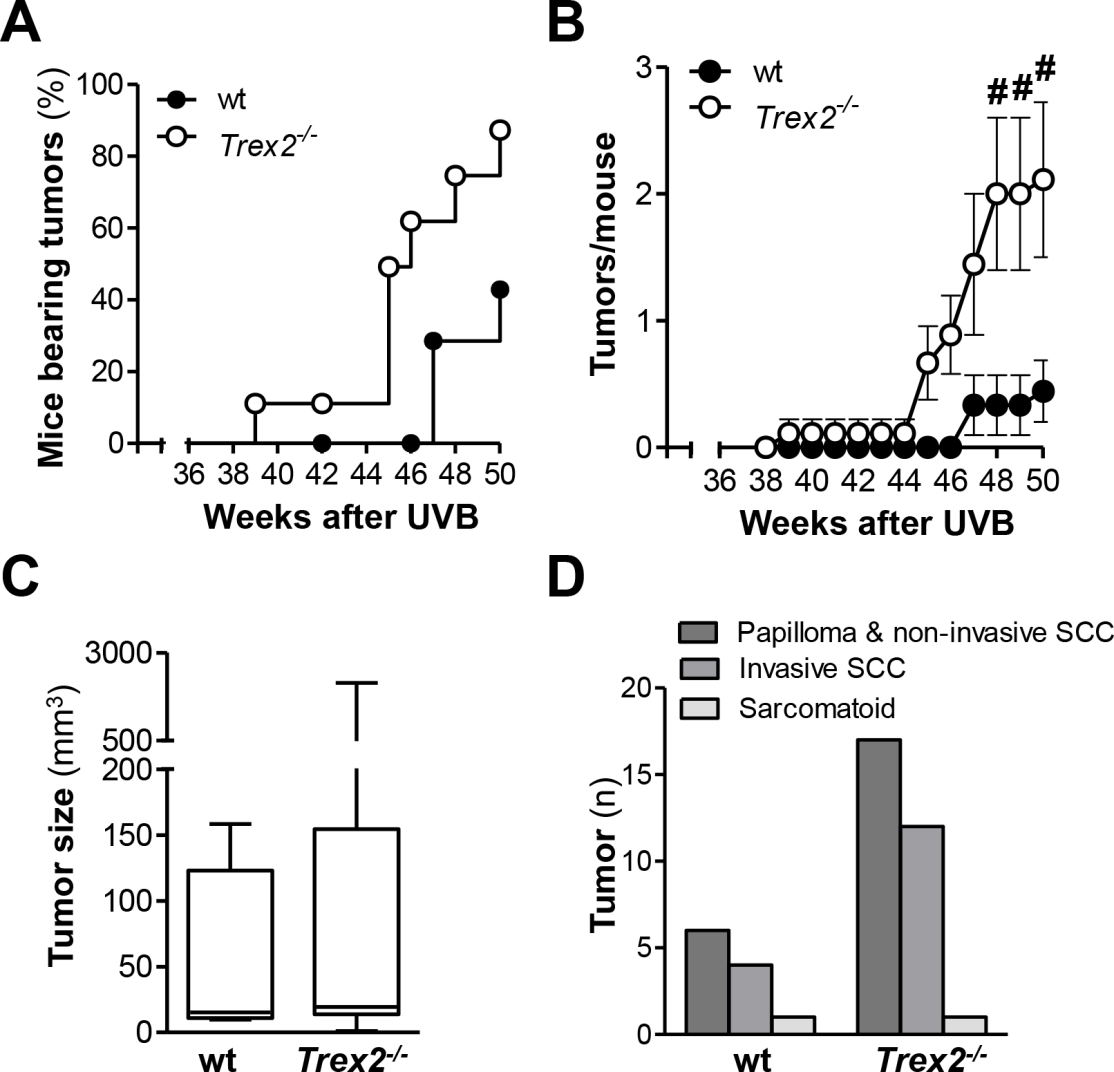
TREX2 deficiency increases susceptibility to UVB-induced skin carcinogenesis Wt (*n* = 9) and *Trex2*^−/−^ (*n* = 9) mice were subjected to the UVB skin carcinogenesis protocol as detailed in Materials and Methods. **A.** Tumor incidence in *Trex2*^−/−^ mice using Kaplan-Meier survival analysis of tumor-bearing mice. **B.** Tumor multiplicity in *Trex2*^−/−^ mice. Average number of tumors *per* mouse. Graphs show mean values and SEM. **C.** Size distribution and **D.** histopathological analysis of skin tumors from UVB-treated wt and *Trex2*^−/−^ mice at the end of the experiment. Significant differences between genotypes: (A) Mantel-Cox test (*P* = 0.025) and (B) Mann-Whitney test (from left to right ^#^*P* = 0.0198, ^#^*P* = 0.0198, and ^#^*P* = 0.0321).

To determine the relevance of TREX2 in UVB-induced skin carcinogenesis, we compared the susceptibility of wt and *Trex2*^−/−^ mice to the development of skin tumors (Fig. 3). The majority of tumors that developed in wt and *Trex2*^−/−^ mice localized to the ear (Supplemental Fig. 2A), but one did form on the dorsal skin of a *Trex2*^−/−^ mouse. *Trex2*^−/−^ mice developed tumors earlier, and tumor incidence was significantly greater in the *Trex2*^−/−^ mice relative to their wt counterparts (Fig. 3A; *P* = 0.025, Log-rank Mantel-Cox Test). By the end of the experiment, 86% of *Trex2*^−/−^ mice had developed tumors, compared to only 43% of wt mice. The average number of tumors per mouse was also significantly higher in *Trex2*^−/−^ mice compared to wt (Fig. 3B), while no differences were seen in tumor size (Fig. 3C), type of tumor and tumor invasiveness (Fig. 3D and Supplemental Fig. 2B) between the two genotypes. Therefore, TREX2 deficiency was associated with increased UVB-induced skin tumorigenesis, supporting a tumor suppressor role for this exonuclease in the skin upon DNA damage.

### TREX2 promotes removal of damaged DNA and keratinocyte cell death in UVB-irradiated skin

**Figure 4:**
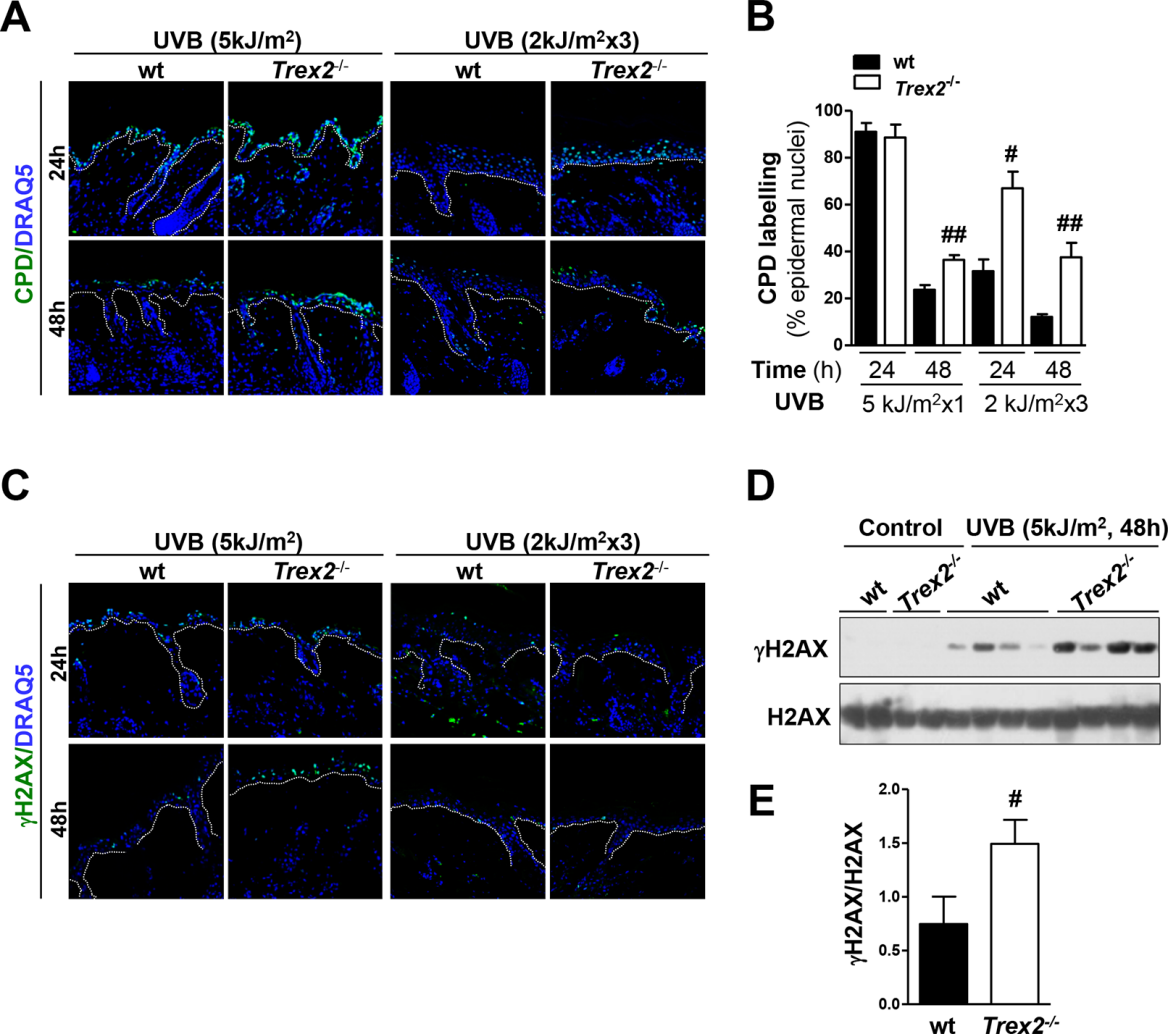
Loss of TREX2 triggers the highest accumulation of UVB-induced DNA damage in the skin **A.** CPD immunostaing and **B.** percentage of CPD-positive epidermal cells and in UVB-irradiated skin from wt and *Trex2*^−/−^ mice. Significant differences between genotypes: Mann-Whitney test (#*P* < 0.05; ##*P* < 0.01). **C.** γH2AX immunostaining, **D.** western blots analysis of γH2AX and H2AX, and **E.** quantification of blotting bands. Values were normalized to the amount of H2AX and are presented as arbitrary units. *P* value is ^#^*P* = 0.035 calculated by unpaired Student's test. Graphs show mean and SEM of at least 4 mice. Nuclei were counterstained with DRAQ5. White dotted lines demarcate the epidermal-dermal border. Original magnification, 40x.

Because the ability of TREX2 to protect the skin against UVB-induced carcinogenesis may reside in both acute and chronic responses to UVB radiation, which acts as both tumor initiator and promoter [17], we examined the effects of TREX2 deficiency on DNA damage removal and cell death following both single and repeated UVB irradiation. Keratinocyte cell death in UV-irradiated skin can take place through diverse cell death modes, including apoptosis and necrosis, depending on doses and frequency of UV irradiation [18]. Immunofluorescence analysis of UVB-induced cyclobutane pyrimidine dimers (CPD) lesions (Fig. 4A-4B) indicated that DNA damage was significantly higher in *Trex2*^−/−^ epidermis 48 hours after acute (5 kJ/m^2^), and at 24 and 48 hours after chronic (2 kJ/m^2^ × 3) UVB irradiation. Additionally, immunofluorescence (Fig. 4C) and western blot (Fig. 4D-4E) analyses of γH2AX, an early chromatin modification at sites of DNA DSBs [19], indicated that by 48 hours after acute UVB exposure the levels of broken DNA were significantly greater in the *Trex2*^−/−^ epidermis compared to wt. Although no obvious differences were observed after chronic UVB exposure, low levels of γH2AX-positive cells complicated the analysis.

**Figure 5:**
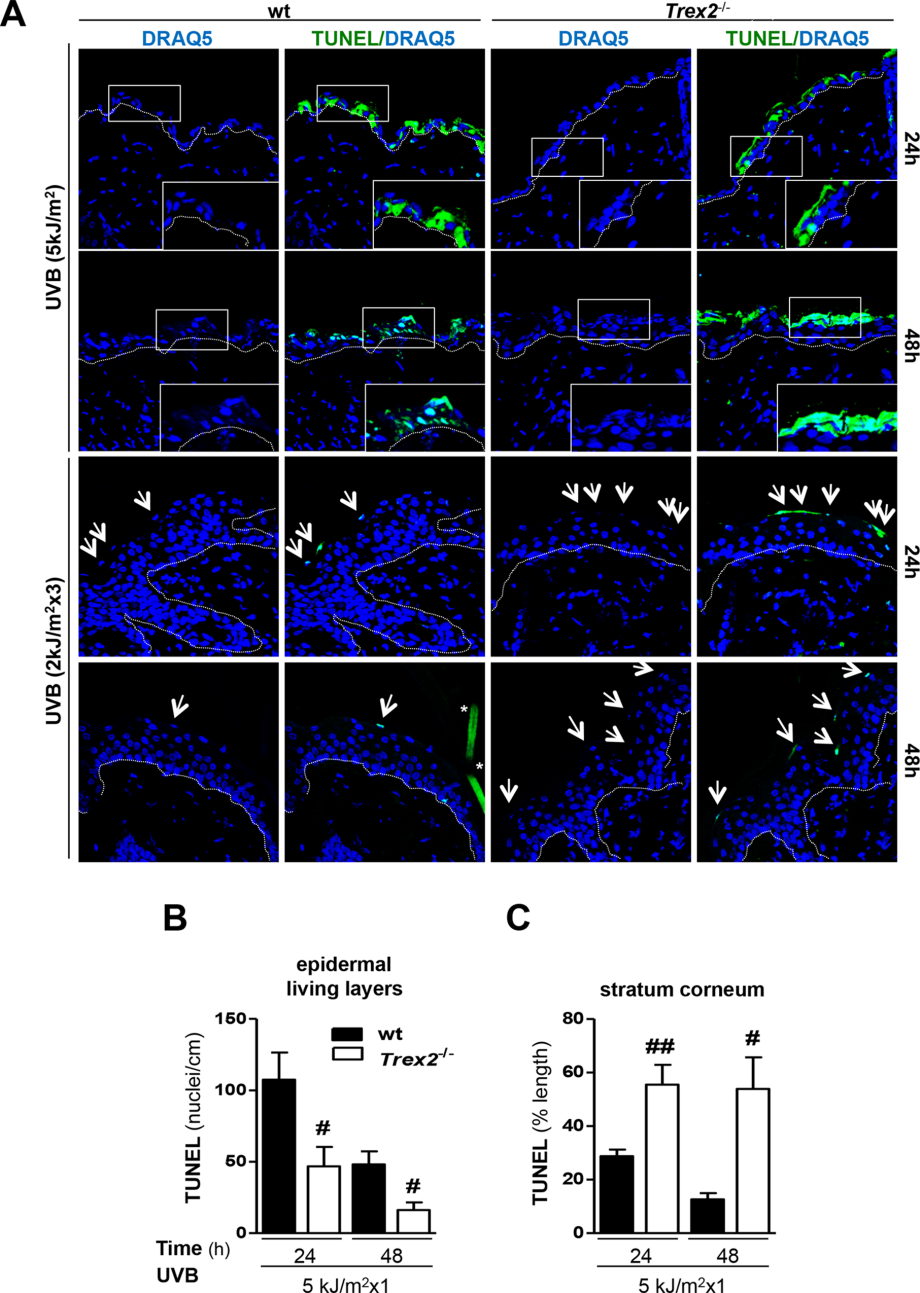
TREX2 is involved in DNA processing and facilitates cell death in UVB-irradiated skin **A.** TUNEL staining in UVB-irradiated skin of wt and *Trex2*^−/−^ mice. Nuclei were counterstained with DRAQ5. White dotted lines demarcate the epidermal-dermal border. Inserts are higher magnifications of boxes. Representative images are shown. Original magnification, 63x. **B.** Quantitative analysis of the UVB-induced TUNEL pattern labeling in the epidermal living layers and **C.** the stratum corneum. TUNEL-positive nuclei number and percentage of lineal TUNEL-labeling pattern are relative to epidermis length. Significant differences between genotypes: Mann-Whitney test (#*P* < 0.05; ##*P* < 0.01). Graphs show mean and SEM of at least 4 mice from each genotype.

Notably, analysis of TUNEL labeling, a marker of DNA strand breaks with free 3′-OH ends that are generated in late-stage cell death modes, including apoptosis and necrosis, and in DNA damaged cells [19, 20], showed that DNA degradation and cell death in damaged keratinocytes were perturbed in the absence of TREX2. TUNEL images illustrated a differential labeling pattern in *Trex2*^−/−^ epidermis compared with that of wt after UVB irradiation (Fig. 5A). There was a significantly lower number of TUNEL-positive nuclei detected in the living layers (stratum basale, spinosum and granulosum) of the epidermis of *Trex2*^−/−^ mice compared with that of wt at 24 and 48 hours after acute UVB irradiation (Fig. 5A-5B), suggesting that loss of TREX2 prevented cell death execution. Conversely, there was a significantly higher amount of TUNEL-positive chromatin after acute and chronic UVB irradiation in the stratum corneum of *Trex2*^−/−^ mice (Fig. 5A-5C), indicating that in the absence of TREX2 fragmented DNA accumulated more in cornified, dead keratinocytes. Consistently, high amounts of TREX2 were observed in TUNEL-positive suprabasal cells from UVB-irradiated wt skin (Supplemental Fig. 3). TUNEL labeling was not detectable in non-irradiated skin of either wt or *Trex2*^−/−^ skin (Supplemental Fig. 4). Thus, these results indicated that TREX2 exonuclease activity contributes to the degradation of DNA in UVB-irradiated epidermal keratinocytes whereas it was redundant under non-stressed conditions.

The relatively reduced amount of TUNEL, γH2AX and CPD positive cells detected after chronic UVB exposure would be related to the lower dose used for chronic (2 kJ/m^2^) compared to acute (5 kJ/m^2^) UVB irradiation. Also, chronic UVB irradiation can lead to acquisition of resistance to apoptosis (Ouhtit et al, 2000). Altogether, these results suggested that TREX2 was involved in the degradation of DNA from dying keratinocytes upon UVB irradiation, and in the absence of TREX2 a fraction of keratinocytes with DNA damage failed to undergo cell death.

### Loss of TREX2 alters inflammatory and immune gene signatures in UVB-irradiated skin

**Figure 6:**
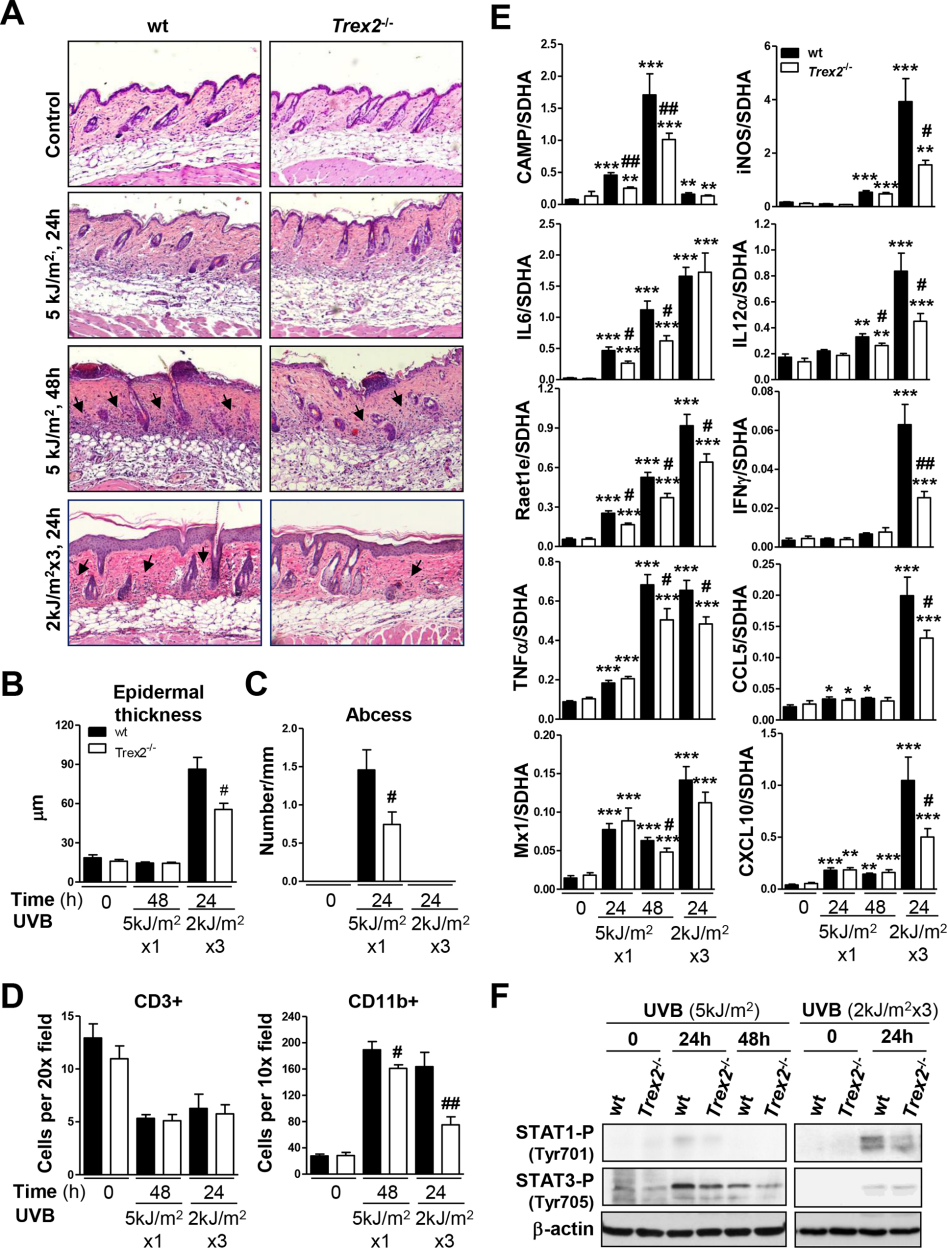
UVB-induced inflammatory response in skin is impaired by the absence of TREX2 **A.** H&E staining of *Trex2*^−/−^ and wt skins showing UVB-induced abscess formation, inflammatory infiltrate and epidermal hyperplasia. Perifollicular immune infiltration (arrows), abscess formation and epidermal hyperplasia are reduced in *Trex2*^−/−^ skin compared to wt. Original magnification was 10x. **B.** Epidermal hyperplasia was evaluated by measuring thickness. **C.** Abscess number is relative to epidermis length. **D.** Inflammatory infiltrate was evaluated by immunostaining of myeloid cells (CD11b+) and T lymphocytes (CD3+) and represented as the mean number of cells counted from at least four images captured at the indicated high-power fields. **E.** Expression of UVB-induced immune genes, as determined by RT-qPCR. Graphs show mean and SEM of at least four mice from each condition and genotype. **F.** Levels of STAT1 and STAT3 tyrosine-phosphorylation in acute and chronic UVB-treated skin of wt and *Trex2*^−/−^ mice were assessed by western blot. Significant differences by Mann-Whitney test between genotypes: #*P* < 0.05, ## *P* < 0.01 and compared to non-irradiated samples: *P < 0.05, **P < 0.01, ***P < 0.001.

The histopathological analysis of UVB-irradiated skin revealed that *Trex2*^−/−^ mice developed less UVB-induced skin inflammation than wt (Fig. 6A). In addition, the UVB-induced increase in epidermal thickness was significantly less pronounced in *Trex2*^−/−^ than in wt mice after chronic UVB exposure (Fig. 6B). Specifically, the relative amounts of epithelial abscesses and dermal inflammatory infiltrates were lower in *Trex2*^−/−^ skin than in wt skin 48 hours after acute UVB irradiation (Fig. 6C). Wt, but not *Trex2*^−/−^ skin, consistently displayed perifollicular immune cell infiltration 24 hours after chronic UVB exposure. At these stages, immunostaining using markers of myeloid cells (CD11b+) and T lymphocytes (CD3+) showed that the extent of UVB-induced myeloid infiltration, which mostly include neutrophils, natural killers and macrophages, was significantly diminished in *Trex2*^−/−^ skin compared to wt, whereas no differences were observed in the UVB-induced depletion of T cells (Fig. 6D, Supplemental Fig. 5A). Therefore, in the absence of TREX2 primary innate immune cell response to UVB was compromised. Notably, although chronic inflammation is generally associated with tumor progression, tumor immunosurveillance is mainly related with early activation of innate immunity that further modulates the adaptive immune response to eventually eliminate transformed cells [18, 21].

Interestingly, the absence of TREX2 impaired induction of several key immune genes by UVB (Fig. 6E), in agreement with the phenotype displaying lower myeloid immune cell infiltration observed in the *Trex2*^−/−^ skin compared to wt. Specifically, the acute UVB-induced up-regulation of IL6, CAMP and Raet1e at 24 and 48 hours, and TNFα and IL12α at 48 hours was significantly lower in *Trex2*^−/−^ skin compared with wt. Similarly, 24 hours after chronic UVB irradiation, increases in the mRNA of TNFα, IL12α, Raet1e, CAMP, IFNγ and the IFNγ-dependent genes CXCL10, CCL5 and iNOS were significantly less in *Trex2*^−/−^ than in wt skin. Consistent with these findings, UVB-induced phosphorylation of STAT1 and STAT3, which are triggered by IFNγ and IL6 signaling, respectively, was reduced in *Trex2*^−/−^ skin compared to wt (Fig. 6F). No significant differences were found in the UVB-induced increase in IL1β, IL1α, IL10, TGFβ, IFNκ, and IRF7 mRNA expression in *Trex2*^−/−^ skin compared with wt (Supplemental Fig. 5B). Collectively, these data highlight the relevance of TREX2 in promoting UVB-induced skin inflammatory and immune responses.

### *Trex2* deficiency impairs DNA repair and hinders cell death in UVB-irradiated keratinocytes

**Figure 7:**
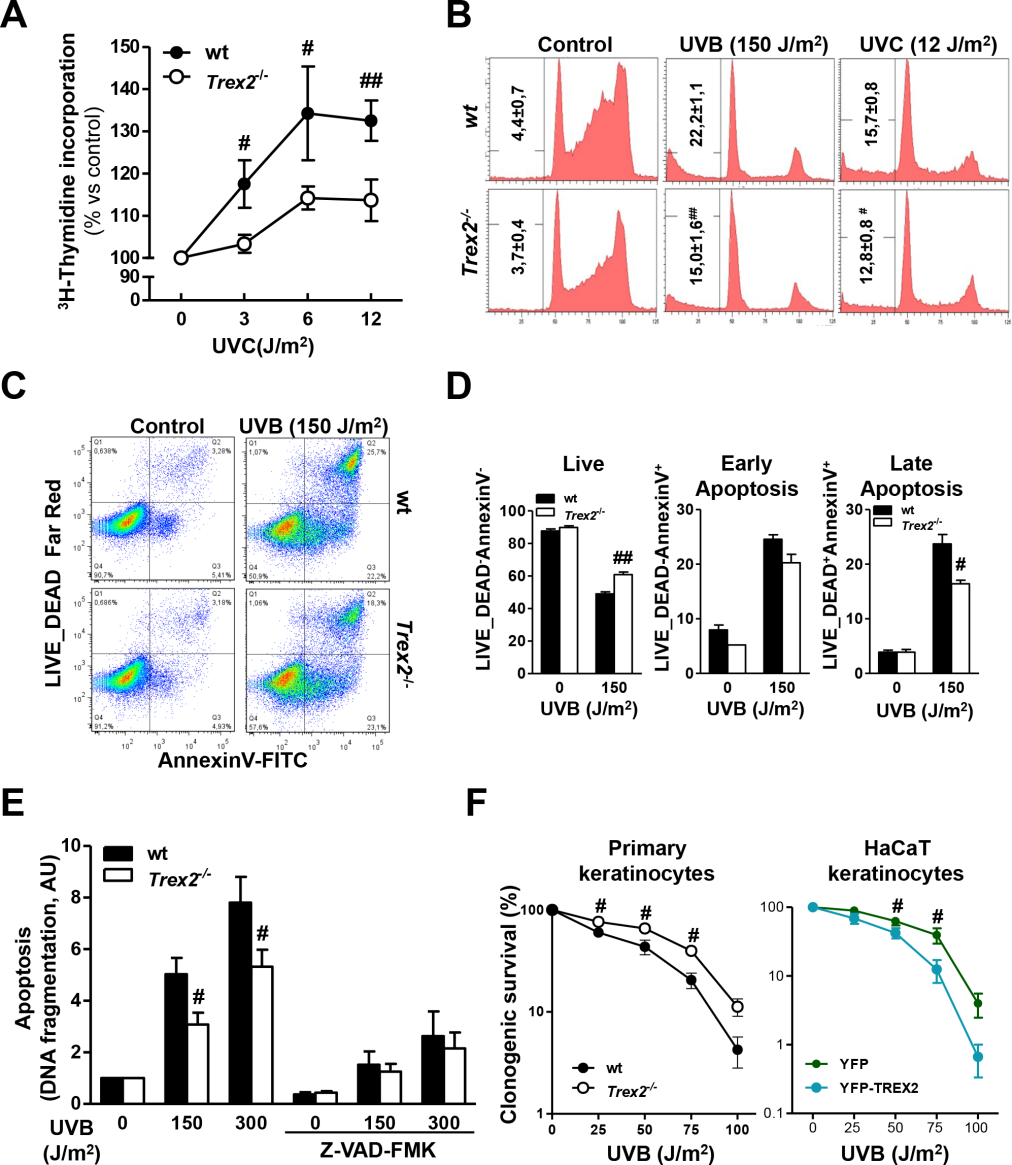
TREX2 deficiency impairs DNA repair and hinders apoptosis in UV-treated keratinocytes **A.** Unscheduled DNA synthesis (UDS) in UVC-irradiated wt and *Trex2*^−/−^ keratinocytes. Data represent percentage of ^3^H-thymidine incorporation over non-irradiated cells. **B.** Cell cycle progression in wt and *Trex2*^−/−^ UV-irradiated keratinocytes. Percentages of cells in the SubG1 phase are indicated. **C.** FACS analysis of Annexin-V and LIVE/DEAD^®^ labeled wt and *Trex2*^−/−^ keratinocytes. **D.** Percentages of unlabelled (live), single-Annexin-V positive (early apoptotic) or double-labeled (late apoptotic) cells are indicated. **E.** UVB-induced DNA fragmentation in wt and *Trex2*^−/−^ keratinocytes. Where indicated, cells were pre-treated with Z-VAD-FMK for one hour. DNA fragmentation is displayed relative to untreated cells. **F.** Keratinocyte survival in the response to UVB. Clonogenic survival of wt and *Trex2*^−/−^ keratinocytes and HaCaT cells expressing YFP or YFP-TREX2 irradiated with the indicated doses of UVB. Graphs show mean and SEM of at least three independent experiments. Images from a representative experiment from three total replicates are shown in B and C. *P* values between genotypes by unpaired Student's *t* test: #*P* < 0.05, ##*P* < 0.01, unpaired Student's *t*-test.

To evaluate the role of TREX2 in the keratinocyte response to UVB while excluding crosstalk with other skin cells, we analyzed DNA repair, cell cycle progression, and apoptosis in UV-treated wt and *Trex2*^−/−^ keratinocytes. Analysis of unscheduled DNA synthesis (UDS) by ^3^H-thymidine incorporation, as a measure of global genome repair [22], revealed that TREX2 deficiency reduced DNA repair synthesis in UVC-treated keratinocytes (Fig. 7A). Similarly, UVB (75 J/m^2^)-treated wt keratinocytes showed DNA repair (108 ± 2, 9%, *n* = 3, over non irradiated cells), but it was absent in *Trex2*^−/−^ keratinocytes (99, 9 ± 3, 4%, *n* = 3, over non irradiated cells). On the other hand, TREX2 deficiency in cultured keratinocytes was significantly associated with a slight but consistent decrease in the percentage of SubG1 apoptotic cells after UVC and UVB irradiation (Fig. 7B). Wt and *Trex2*^−/−^ keratinocytes displayed similar cell cycle progression after exposure to UVC and UVB with both inducing G1 phase checkpoint arrest (Fig. 7B). Interestingly, FACS analysis showed that TREX2 deficiency hindered the passage of UVB-irradiated keratinocytes to late (Annexin-V and Live_Dead double-positive) non-reversible apoptotic stages (Fig. 7C-7D). Consistently, release of nucleosomes into the cytoplasm, a characteristic feature of DNA fragmentation associated with cell death by apoptosis, was reduced in UVB-treated *Trex2*^−/−^ keratinocytes compared to wt (Fig. 7E). No differences in nucleosome release were observed between the two genotypes when caspases were inhibited by Z-VAD-FMK (Fig. 7E), suggesting that TREX2 acts downstream of caspases facilitating DNA degradation in UVB-treated keratinocytes.

Indeed, data from survival colony assays indicated that *Trex2*^−/−^ keratinocytes were more resistant to UVB than were wt (Fig. 7F). In agreement, stable expression of exogenous YFP-TREX2 in HaCaT cells increased the susceptibility to UVB radiation when compared to cells expressing YFP as a control.

Collectively, these results indicated that TREX2 in a cell-autonomous manner played critical roles in promoting keratinocyte apoptosis over DNA repair and cell survival upon UV irradiation.

### TREX2 accumulates in low-density chromatin regions and in micronuclei

**Figure 8:**
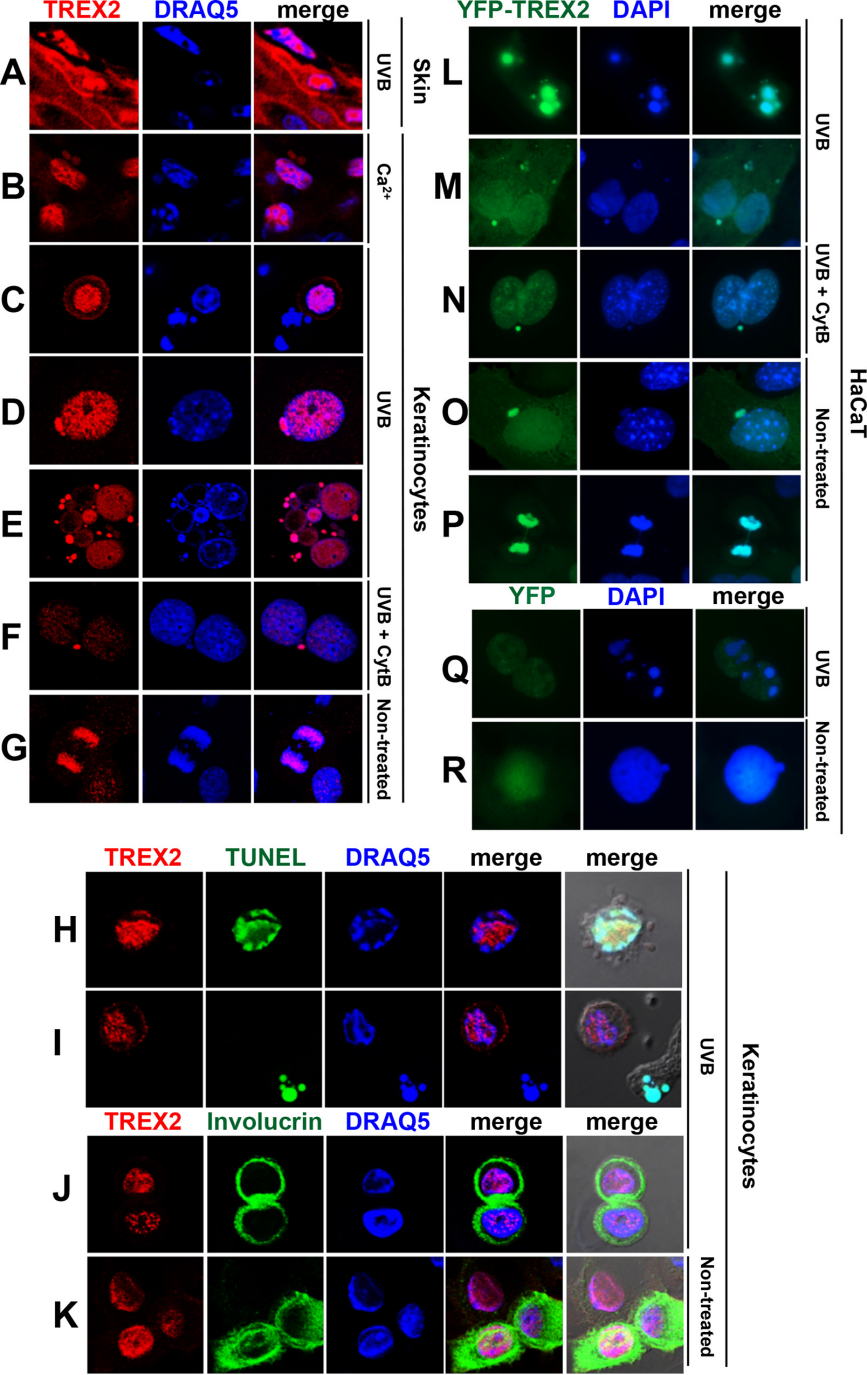
TREX2 accumulates in regions of low-density chromatin and in micronuclei **A.** Mouse skin exposed to chronic UVB (2 kJ/m^2^) radiation, **B.** Ca^2+^-treated mouse keratinocytes, **C-E; H-J.** mouse keratinocytes exposed to UVB (150 J/m^2^) or **F.** to Cytochalasin B (Cyt-B) prior to UVB, and **G.** non-treated keratinocytes undergoing mitosis or **K.** differentiation were stained with TREX2 and involucrin antibodies and TUNEL, as indicated. **L-P.** HaCaT cells stably expressing ectopic YFP-TREX2 or **Q-R.** YFP were (L, M and Q) exposed to UVB (150 J/m2), or to (N) Cyt-B prior to UVB or (O, P and R) left untreated. Nuclei were counterstained with DRAQ5 or DAPI, as indicated. Representative images were shown. Original magnification was 63x (A-K) and 100x (L-R)

TREX2 accumulated in skin suprabasal keratinocytes and, interestingly, in the vicinity of condensed nuclear DNA (Fig. 8A). Also, in primary keratinocytes cultures, upon Ca^2+^ addition (Fig. 8B), which triggers keratinocyte differentiation, or UVB irradiation (Fig. 8C-8F, 8H-8J), the immunofluorescence labeling revealed that multiple, discrete TREX2 foci were present in areas of low-density chromatin in nuclei that were beginning to condensate chromatin while undergoing cell death, which was indicated by TUNEL-positive staining (Fig. 8H-8I). In contrast, TREX2 was not detected in chromatin bodies of late apoptotic cells (Fig. 8C, 8I), suggesting a transient action of TREX2 during apoptosis progression. Moreover, an intense nuclear staining pattern of TREX2 was observed in detaching involucrin-positive keratinocytes committed to cell death via differentiation (Fig. 8J, 8K). Interestingly, strong TREX2 labeling was observed in micronuclei of either single or multinucleated keratinocytes (Fig. 8D, 8E). Notably, the size and density of TREX2 foci in micronuclei of binucleated cells after treatment with the cytokinesis inhibitor cytochalasin-B (Cyt-B) was higher than in the original nuclei (Fig. 8F), suggesting that TREX2 was accumulated in chromatin that was not incorporated into daughter nuclei during mitosis. In fact, TREX2 was also present in mitotic chromatin (Fig. 8G). Accordingly, YFP-TREX2 (Fig. 8L-8P), but not YFP (Fig. 8Q-8R), ectopically expressed in HaCaT cells was abundantly localized in apoptotic, micronuclear and mitotic chromatin, relative to nucleosomal chromatin. Thus, these data suggest that TREX2 could be differentially recruited to distinct chromatin states associated to DNA degradation during keratinocyte death and differentiation.

### TREX2 is recruited to micronuclear damaged DNA, interacting with γH2AX

**Figure 9:**
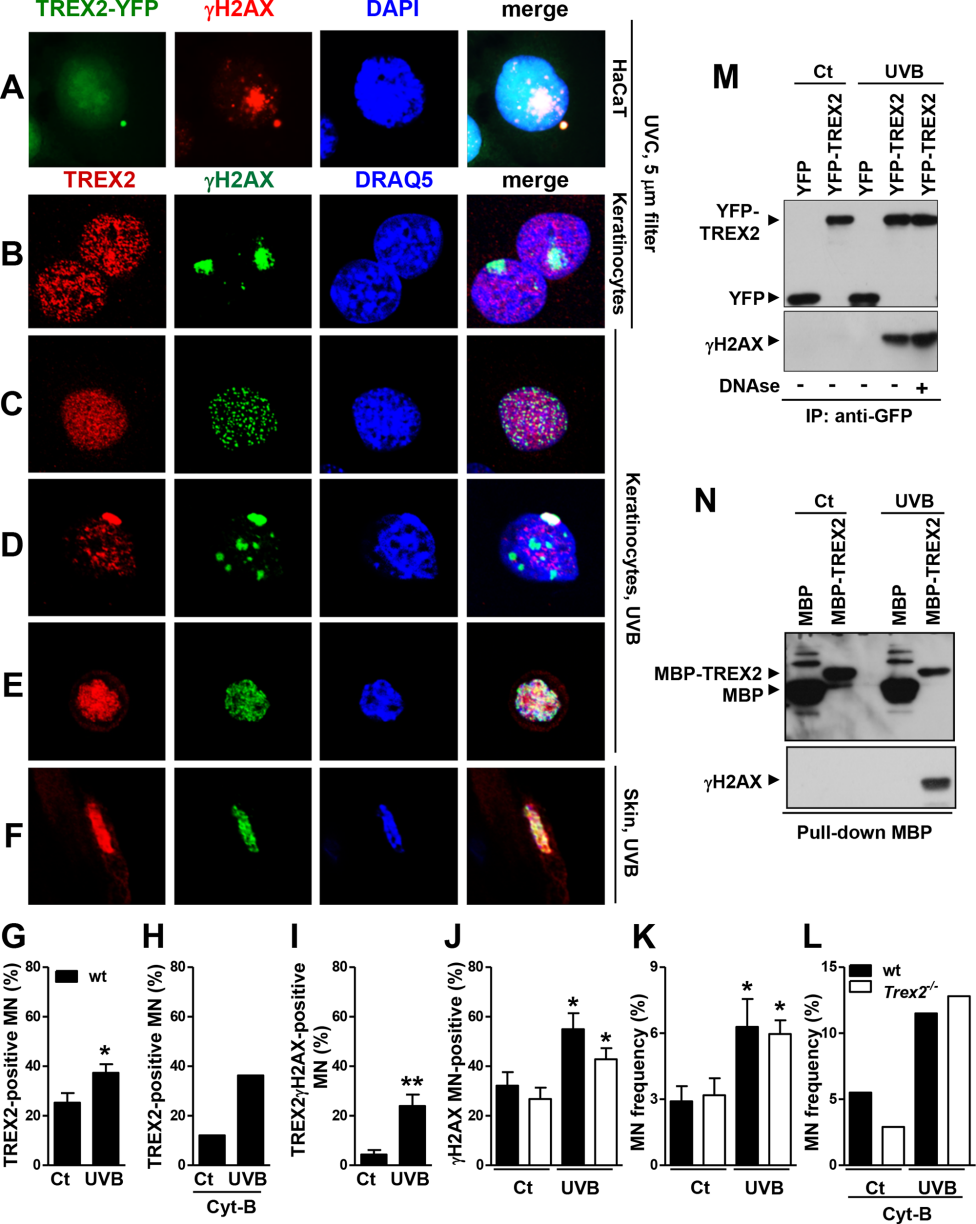
TREX2 co-localizes and interacts with γH2AX in micronuclei **A.** HaCaT cells expressing exogenous YFP-TREX2 and **B.** primary keratinocytes UVC-irradiated (100 J/m^2^) through 5-μm micropore filters, **C.** keratinocytes globally-irradiated with UVB (150 J/m^2^), **D.** containing micronuclei or **E.** detaching, and **F.** UVB-treated skin samples were stained with γH2AX and TREX2 antibodies, as indicated. **G.** Percentage of TREX2 single-positive micronuclei scored in the absence and **H.** in the presence of Cyt-B, and **I.** micronuclei double-positive for TREX2 and γH2AX or **J.** single-positive for γH2AX. **K.** Micronuclei frequency in untreated or UVB-irradiated (150 J/m^2^) wt and *Trex2*^−/−^ keratinocytes in the absence or **L.** presence of Cyt-B. In the presence of Cyt-B, only binucleated cells were scored. The data represent the mean and SEM of three independent experiments (G, I, J, K), and the mean of one (H, L) analysis of up to 1, 000 cells. **M.** TREX2 interacts with γH2AX as shown by coimmunoprecipitation of γH2AX and YFP-TREX2 after UVB (150 J/m^2^) irradiation of HaCaT cells stably expressing YFP-TREX2 and by **N.** MBP pull-down assays. Nuclei were counterstained with DRAQ5 or DAPI, as indicated. Original magnification was 100x (A) and 63x (B-F) *P* values between untreated and UVB-treated keratinocytes calculated by unpaired Student's *t* test: *P < 0.05, **P < 0.01.

To assess whether TREX2 was distributed at sites of damaged DNA, we performed confocal microscopy analysis of TREX2 and γH2AX immunostaining. In UVC-irradiated cells through 5 μm micropore filters, TREX2 was not recruited to nuclear sites of localized γH2AX formation (Fig. 9A-9B), whereas Rad51 accumulated at sites of damaged DNA (Supplemental Fig. 6) as previously described [23]. We only detected overlap of TREX2 with a few nucleosomal γH2AX foci in globally UVB-irradiated keratinocytes (Fig. 9C). Interestingly, abundant TREX2 co-localization with uniformly labeled γH2AX was frequently observed in micronuclei (Fig. 9A, 9D). Importantly, γH2AX is required for DNA degradation during cell death [24] and homogeneous γH2AX labeling affecting all micronuclear chromatin is associated with its specific degradation independent of nuclear degradation [25]. In this context, UVB radiation triggered a significant increase in the frequency of micronuclei that were TREX2 positive (Fig. 9G, 9H), γH2AX positive (Fig. 9J) and γH2AX-TREX2 double positive (Fig. 9I). However, no significant differences were observed in the frequency of basal or UVB-induced micronuclei (Fig. 9K-9L) or γH2AX-positive micronuclei (Fig. 9J) between wt and *Trex2*^−/−^ keratinocytes, indicating that TREX2 is not involved in micronuclei formation. Nevertheless, we cannot exclude that micronuclear DNA content differs between the two genotypes. Furthermore, substantial co-localization of TREX2 and γH2AX occurred in detaching keratinocytes in culture (Fig. 9E) and skin suprabasal keratinocytes (Fig. 9F) undergoing UVB-induced cell death. Therefore, TREX2 and γH2AX co-localization seemed to be associated with DNA degradation rather than DNA repair.

Indeed, co-immunoprecipitation assays demonstrated that TREX2 interacted with γH2AX in UVB-treated HaCaT cells overexpressing YFP-TREX2 protein (Fig. 9M). This interaction was independent of its binding to DNA since was not abrogated by DNAse treatment of cell extracts prior to co-immunoprecipitation assay. In addition, γH2AX associates with TREX2, by MBP pull-down assays (Fig. 9N). These observations indicate that TREX2 could gain access to DNA ends by interacting with γH2AX.

## DISCUSSION

**Figure 10:**
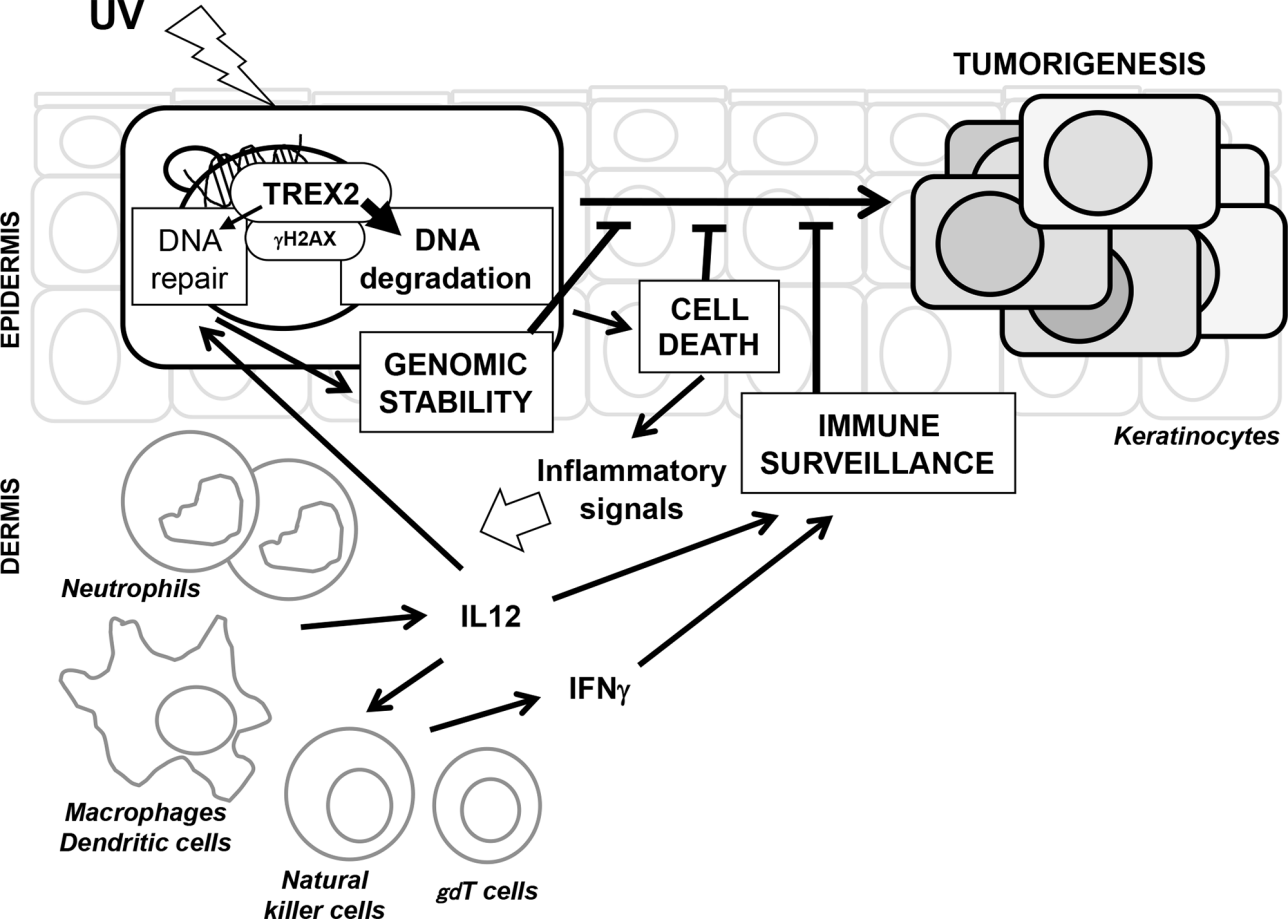
A model for the molecular events of TREX2 in UV-irradiated skin. UV irradiation causes DNA damage in epidermal keratinocytes. TREX2 contributes to DNA repair which facilitates the survival of cells with limited damage In severely damaged cells, TREX2 is active in DNA degradation during cell death, leading to inflammatory signals that subsequently activate innate immune cells. The immune regulatory cytokine response is associated with increased DNA repair capacity and immune surveillance of the epidermis. The dual effect of TREX2 is, at least partly, mediated by its interaction with γH2AX, a critical player of the DNA damage response that displays a critical role in both DNA repair and cell death execution. Loss of TREX2 function leads to increased keratinocyte genomic instability and impaired skin immune surveillance after UV irradiation and ultimately to carcinogenesis.

Altogether, our results show that in the skin response to UVB radiation, TREX2, an exonuclease specifically expressed in keratinocytes, regulates keratinocyte cell death and, as a result, skin inflammatory and immune responses, that are crucial to counteract carcinogenic effects of the UV radiation. In damaged keratinocytes, TREX2 intrinsically affects DNA degradation rather than DNA repair, prompting cell death execution and subsequently driving a skin immune response associated with increased DNA repair and immune surveillance. Interestingly, we discovered that TREX2 interacts with γH2AX, a critical player in DNA repair that is also required for DNA degradation in either apoptosis or necrosis cell death modes [24]. A model for the role of TREX2 in UV-irradiated skin is depicted in Figure 10.

TREX2 deficiency increases skin carcinogenesis induced by chemical [6] or UVB radiation (Fig. 3A). In support of a relevant tumor suppressor role for TREX2 in squamous tumorigenesis, we observed alterations in the *TREX2* gene and TREX2 expression in human SCCs. Similar to NM23-H1, a tumor suppressor that also possess 3′-5′ exonuclease activity [26], TREX2 is highly expressed in premalignant lesions, but is lost with tumor dedifferentiation. Regarding *TREX2* gene, our data confirms that somatic mutations are not among the diverse genetic alterations that accumulate in HNSCC [27], but the slightly higher incidence of rare *TREX2* variations in SCC patients suggests that genetic variability might be a predisposing factor for the onset of carcinogenesis. To date, only a few rare SNPs in nucleotide excision repair (NER) and Fanconi anemia genes have been associated with susceptibility to squamous carcinogenesis [28, 29]. Interestingly, the TREX2 R156L variant, which results in a significant loss of function, presents also a slightly higher frequency in sporadic prostate cancers [30]. In this context, a germline TREX2 inactivating mutation has been reported in one colorectal cancer patient [31]. Further evaluation of large cohorts should provide insights into a putative association of *TREX2* variants with SCC susceptibility.

The results showed here support that upon UV irradiation, TREX2 facilitates both DNA repair and cell death. However, the pro-apoptotic activity of TREX2 dominates its pro-survival activity, highlighting a critical role for TREX2 in DNA damaged-induced cell death. Our data indicate that TREX2 does not participate to a significant degree in the NER pathway, which is responsible for removing primary UV-induced lesions. This is consistent with a lack of contribution of 3′-5′ exonuclease activity to NER molecular mechanisms [32, 33]. On the other hand, ectopic co-expression of TREX2 with the i-Sce1 endonuclease has demonstrated that degradation of DNA 3′-ends by TREX2 exonuclease activity avoids restoration of the i-Sce1 site after end joining repair [8]. Indeed, TREX2 can paradoxically modify DNA repair via EF-PRR and NHEJ pathways either in a positive or negative way [8, 10], and its prevalence would most likely be determined by the type of DNA lesion and cellular context. For instance, there is a significant diversity in the DNA repair and apoptosis responses to UV among keratinocytes and fibroblasts [34]. Our data clearly indicate that in primary keratinocytes, TREX2 promotes apoptosis progression to late stages, when the death of keratinocytes carrying DNA breaks is ensured. Analogously, the nonprocessive 3′-5′ exonuclease TREX1 has been shown to be involved in DNA degradation from the 3′-ends generated by the NM23-H1 endonuclease during granzyme A-mediated cell death [35]. Importantly, TREX2 interacts with γH2AX, which plays a critical role in both DNA repair and DNA degradation [24, 36], in the context of the chromatin-degrading state that is present in micronuclei and nuclei of keratinocytes undergoing cell death via apoptosis, necrosis or differentiation programs [13, 25, 37]. It is therefore tempting to speculate that the TREX2-γH2AX interaction might be part of the DNA degradosome [24], thereby participating directly in DNA degradation once 3′-ends are available in cells undergoing DNA damage-induced cell death.

UV radiation elicits complex cellular signaling and keratinocyte-immune cell crosstalk that leads to coordinated immune response to maintain skin homeostasis and immune surveillance against cancer [15, 16]. Danger signals such as self-DNA from stressed and dying keratinocytes trigger the production of type I interferons and pro-inflammatory cytokines [38]. Unexpectedly, TREX2 deficiency reduces inflammation and does not lead to a differential type I interferon response signature. This is in spite of the highest accumulation of fragmented DNA in corneocytes from *Trex2*^−/−^ skin. Most likely, DNA in the stratum corneum becomes inaccessible to skin plasmacytoid dendritic cells, which are the major type I IFN producers after sensing DNA fragments. In contrast, signals from dying keratinocytes, more abundant in wt than *Trex2*-deficient skin upon UVB exposure, can be easily sensed by dendritic and natural killer cells, which are responsible for secretion of major pro-inflammatory cytokines. Importantly, our findings reveal that TREX2 drives an immunoregulatory cytokine signature associated with increased DNA repair and anti-tumorigenic functions. Specifically, loss of TREX2 leads to reduced UVB-induction of genes that play a pivotal role in skin immune surveillance, such as IL12, IFNγ, CXCL10, and Raet1e [39, 40]. In fact, IL12 is required for UV-induced DNA damage repair in skin through NER [41]. The highest levels of DNA damage in *Trex2*-deficient skin after UVB exposure could be attributed to the lowest induction of this cytokine. Notably, IL-12 and IFNγ are well-known drivers of cytotoxic T cells, which inhibit initiation and progression of skin cancer [21]. Furthermore, decreased up-regulation of IL6 and TNFα, pro-inflammatory cytokines that regulate keratinocyte proliferation and cell death [42, 43], correlates with the reduced apoptosis, hyperplasia and inflammation observed in *Trex2*-deficient skin following UVB exposure. Therefore, TREX2 positively regulates the inflammatory response in contrast to the negative role of the closely related TREX1 exonuclease [44, 45], supporting the specific and non-redundant roles of these exonucleases as key regulators of inflammation. TREX1 plays an essential role in immune silencing of DNA by degrading endogenous cytosolic ssDNA [46], retroelements [47], and unmodified DNA [48] during normal homeostasis. In addition, unlike TREX2, TREX1 protects against cell death following UVC radiation [49]. An intriguing question is how TREX2 triggers UVB-induced positive inflammatory function. This may be related to its activity promoting cell death. Damage-associated molecular patterns, including modified nucleic acids, released from dead cells [18] elicit activation of innate immune receptors leading to a pro-inflammatory response [38]. Also, TNFR1-induced keratinocyte apoptosis leads to skin inflammation [50, 51]. Furthermore, because short DNA fragments induce stronger inflammatory response than long fragments do [52], it is tempting to hypothesize that TREX2 can contribute to shorter DNA fragments, thus favoring DNA sensing rather than immune silencing. Further insight into the mechanisms by which TREX2 regulates skin immunity under stress conditions may have major implications in the understanding of inflammatory skin diseases. Our findings suggest that TREX2 can be part of relevant keratinocyte defense mechanisms against UVB and other environmental mutagenic threats or stressors.

## MATERIALS AND METHODS

### Mice and UVB treatments

The *Trex2*^−/−^ mouse has been described previously [6]. *Trex2*^−/−^ and wt C57BL/6 mice were housed in a specific-pathogen-free environment according to the requirements of the Ethics Committee on Animal Care of Parc Científic of Barcelona. All experiments were conducted under the authorization of the Catalan Government (DAAM 7067). The UVB light source was from a CL-1000M Midrange Ultraviolet Crosslinker (UVP Inc.) that emitted UVB light at a wavelength of 302 nm. UVB treatments were applied to the ears and back skin, previously shaved with electric clippers, of 8 to 10 week-old female mice. For the photocarcinogenesis study, we followed an established protocol with minor modifications [53]. Briefly, mice from each genotype were irradiated three times per week for 12 weeks with UVB at a dose of 2 kJ/m^2^ per exposure, followed by the next 12 weeks with 3 kJ/m^2^ per exposure and 5 kJ/m^2^ per exposure for the final 26 weeks. Mice were visually inspected weekly, and tumor numbers and sizes were evaluated to calculate tumor incidence (percentage of tumor-bearing mice) and tumor multiplicity (average number of tumors per mouse). Mice were sacrificed when they showed signs of poor health. For the studies on the short term effects of UVB, wt and *Trex2*^−/−^ mice were evaluated in two experimental stages: (i) 24 and 48 hours following exposure to a single dose of UVB (5 kJ/m^2^), and (ii) 24 and 48 hours after the last exposure to repeated UVB exposures (2 kJ/m^2^) on three non-consecutive days. These stages are referred to as acute and chronic UVB exposure, respectively. Control mice were shaved but received no UVB exposure. These experiments were conducted and repeated in 4 to 7 individual animals of each genotype per experimental group. Skin samples were collected for histological evaluation and RNA and protein analyses.

### Human samples

Cutaneous SCC (cSCCs) [54] and HNSCC [55] tissue microarrays (TMAs) have been described previously. DNA samples from patients diagnosed with an HNSCC and those from healthy controls were obtained from the Banco Nacional de ADN (University of Salamanca, Spain). Approval was obtained by the ethics committees from all participating centers and in accordance with the guidelines of the Helsinki Declaration.

### TREX2 genotyping in HNSCC

DNA extraction from blood and tumor samples with quality controls was performed by Banco Nacional de ADN following standard procedures. The PCR products of the 3265 bp region (X Chromosome: 152710145-152713410) covering the entire *TREX2* coding sequence and un-translated regions were directly sequenced. PCR reactions were performed using the Expand High Fidelity PCR system (Roche) in 50 μl reaction volumes containing 50 ng genomic DNA and 10 μM primers (sequences are available upon request). Purified PCR products (Pure Link PCR purification kit, Life Technologies) were sequenced on both strands using the BigDye Terminator v3.1 Cycle Sequencing Kit and analyzed on an ABI prism 3730 (Applied Biosystems).

### TREX2 variant generation and exonuclease activity assays

Human TREX2 variants were generated by site-directed mutagenesis, cloned into the pLM303x plasmid and then purified as described [56]. The R156L variant was previously generated in a plasmid construct that left six fewer residues at the N-terminus compared to the constructs used for the other variants, resulting in a lower molecular weight. The ssDNA and dsDNA exonuclease activities were assayed as previously described [56]. For ssDNA exonuclease activity, standard reactions were prepared with a fluorescein-labeled 30-mer oligonucleotide and dilutions of the recombinant TREX2 variants were prepared at 10 times their final concentrations. Samples containing the TREX2 enzymes were added to the reactions to yield the final indicated concentrations. The incubations were 30 min at 25°C. The reaction products were then subjected to electrophoresis on 23% urea-polyacrylamide gels and quantified. For dsDNA exonuclease activity, titration and time course reactions were prepared with nicked-dsDNApMYC plasmid. Reactions were performed at the designated concentrations for 30 min and the reaction products subjected to electrophoresis on agarose gels and visualized by ethidium bromide staining. Time course reactions were performed with 200 nM of each TREX2 variant and samples were removed prior to addition of the enzyme (0 min) and at the indicated incubation times. The reaction products were quenched in 15x SYBR Green II, and remaining dsDNA was determined by emission at 520 nm. The plot of TREX2 activity was generated using SigmaPlot 8.02 (SPSS Science, Inc.).

### Cell lines and UV treatment, apoptosis, cell cycle and survival assays

Primary keratinocytes were prepared from newborn mouse skin and cultured in CNT-02 medium (CELLnTEC) for up to seven days, as previously described [6]. Immortalized HaCaT keratinocyte cells stably expressing exogenous YFP (HaCaT_YFP) or YFP fused to mouse TREX2 (HaCaT_YFP-TREX2) and grown in DMEM supplemented with 10% FBS, were selected with G418 (100 μg/mL; Sigma) and sorted by flow cytometry (MoFlo Astrios, Beckman Coulter).

Cells were exposed to UV irradiation after removal of the medium using UVB (UVP Inc.) or UVC (Sankyo Denki) lamps at a distance of 50 cm, followed by the addition of fresh medium. A radiometer with UVX-25 and UVX-31 sensors was used (UVP Inc.) to monitor UV dose. Wt and *Trex2*^−/−^ keratinocytes were either untreated or irradiated with UVB or UVC at the indicated doses. To measure UDS (Lehmann & Stevens, 1980), keratinocytes were cultured in CNT-02 medium without growth factor supplements and with 20 mM hydroxyurea for 3 hours to inhibit semi-conservative DNA replication. The cells were then UV-irradiated and incubated for an additional 3 hours in the same fresh media containing 20 μCi/ml ^3^H-thymidine. Incorporation of ^3^H-thymidine into acid-insoluble materials was measured by liquid scintillation counting.

UV-induced cell death was assessed 18 hours post-irradiation and evaluated to detect DNA fragments released into the cytoplasm using the Cell Death Detection ELISA^PLUS^ kit (Roche) according to the manufacturer's protocol. Progression of apoptosis was determined with the Annexin V-FITC Apoptosis Detection kit (eBioscience) and the LIVE_DEAD-Far Red reagent (Life Technologies). Cells were stained prior harvesting to avoid keratinocyte membrane damage by trypsin. The stained cells were analyzed by flow cytometry (FACSCanto II, Becton Dickinson). Annexin-positive cells corresponded to early apoptotic cells while Annexin-positive plus strong positive-LIVE_DEAD-Far Red cells corresponded to late apoptotic cells.

To determine the effects of UVB radiation on cell survival, clonogenic assays were performed. Cells (2, 5# × # 10^5^ primary keratinocytes or 10, 000 HaCaT_YFP and HaCaT_YFPTREX2) were seeded on 6-well plates in duplicate, and treated with the indicated doses. Ten days after treatment, the colonies were stained with methylene blue following standard procedures, scored and used to calculate the surviving fraction of cells in each condition.

Cell cycle was assessed 18 hours post-irradiation. Fixed cells were stained with propidium iodide (Sigma) following a standard procedure and analyzed by flow cytometry (FACSCanto II, Becton Dickinson). For cell cycle progression analysis, debris and residuals of necrotic cells were gated off during the analysis of data, as they have minimal DNA fluorescence and reduced diameter compared to apoptotic cells [57].

Micronuclei were evaluated in the presence and absence of Cytochalasin B (Cyt-B, 0.5 μg/mL, Sigma) to block cytokinesis. In this assay, only binucleated dividing cells were taking into account for micronuclei scoring [58].

For keratinocyte immunofluorescence assays, cells grown on collagen-coated glass coverslips were left untreated, globally irradiated or irradiated through a filter with 5 μm pores.

### Histological and immunofluorescence analyses

Skin and tumor samples from mice were fixed with 4% paraformaldehyde in PBS, embedded in paraffin and then cut in 5 μm sections. Sections were stained with hematoxylin and eosin (H&E) for histopathological evaluation. Tumors were grouped as papillomas and non-invasive SCC, invasive SCC, and sarcomatoid tumors.

Epidermal thickness in H&E stained sections was measured as the distance, in the interfollicular epidermis, between the top of the basement membrane and the bottom of the stratum corneum at 4 points of 2 randomly selected 20x fields from each animal using the Image J software (NIH).

For skin immunostaining analyses, indirect immunofluorescence and immunohistochemistry standard procedures were used. Skin sections were deparaffinized and rehydrated, and antigen retrieval was performed in citrate buffer (pH 6.0) for CPD staining or Tris-EDTA buffer (pH 9.0) for TREX2, involucrin, K10, CD3, CD11b and γH2AX staining. The following primary antibodies were used: affinity-purified rabbit anti-mouse TREX2 (1/50; in house [6]), mouse anti-K10 (1/200; ab9026, Abcam), mouse anti-involucrin (1/200; I9018; Sigma) rabbit anti-CD3 (1/200; ab5690, Abcam), rabbit anti-CD11b (1/200; ab75476, Abcam);) mouse anti-CPD (1/500; COSMO BIO CO), and mouse anti-γH2AX (Ser139) (1/500; Upstate) antibodies. For immunofluorescence, goat anti-mouse IgG coupled to Alexa Fluor 488 and goat anti-rabbit IgG coupled to Alexa Fluor 555 (1/500; Life Technologies) were used as secondary antibodies. Image J software (NIH) was used to quantify the percentage of positive-labeled cells in at least four fields selected randomly from each section in a blinded manner.

Similarly, TMAs of cSCC were processed with Tris-EDTA buffer (pH 9.0) for indirect immunofluorescent detection of TREX2 and involucrin by rabbit anti-human TREX2 (1/100; in house) and mouse anti-involucrin (1/200; Sigma) antibodies. 3-μm sections of HNSCC TMAs were deparaffinized and treated using the Envision Flex Target Retrieval solution low pH. Incubation with rabbit anti-human TREX2 (1/100; in house) antibody was done on an automatic staining workstation (DakoAutostainer Plus) using the DakoEnVision Flex + Visualization System. Samples were counterstained with hematoxylin. For analysis of TREX2 expression in SCCs, immunostaining was independently evaluated by two observers in a blinded manner, and data were dichotomized as negative-weak expression (score 0) *versus* moderate-strong expression (score 1) either in the tumor epithelia or the stroma.

For keratinocyte staining, cells were fixed with 4% paraformaldehyde in PBS and permeabilized with 0.1% Triton X-100 in PBS. The samples were then blocked and incubated with affinity purified rabbit anti-mouse TREX2 and anti-Rad51 (1/100; Santa Cruz Biotechnologies), and mouse anti-γH2AX, anti-involucrin, and anti-CPD antibodies as described above. For anti-CPD labeling, after permeabilization, cells were treated with 2 M HCL for 30 min to denature DNA. Goat anti-mouse IgG coupled to Alexa Fluor 488 and goat anti-rabbit IgG coupled to Alexa Fluor 555 (1/500; Life Technologies) were used as secondary antibodies.

Cell and tissue TUNEL-labeling using the In Situ Cell Death Detection Kit Fluorescein (Roche) was performed prior to antibody incubation and following the manufacturer instructions. TUNEL and epidermis length were quantified along at least 1 cm section using Metamorph software (Molecular Devices, Sunnyvale) in a blinded manner and the average of the images calculated.

Nuclei and micronuclei were counterstained with DRAQ5 (Biostatus Limited) or DAPI (Sigma). Slices were mounted in ProLong Gold Anti-Fade reagent (Life Technologies).

### Microscopic imaging

Confocal images were obtained using a Leica TCS-SL filter-free spectral confocal laser scanning microscope (Leica Microsystems, Germany). Conventional images were captured with a Nikon Eclipse E-800 fluorescence microscope.

### Quantitative RT-PCR analysis

Total RNA isolated from pulverized skin (NucleoSpin RNA extraction kit, Macherey-Nagel) was used for cDNA synthesis (GeneAmp RNA PCR kit, Applied Biosystems). Quantitative PCR amplification reactions were performed in the 7500 Fast Real-Time PCR System (Applied Biosystems). TaqMan assays (Applied Biosystems) were used to quantify the mRNA expression of mouse TREX2 (Mm04210320_m1), TNFα(Mm00443258_m1), IL6 (Mm00446190_m1), IL1β (Mm00434228_m1), IL1α (Mm00439620_m1), IL10 (Mm00439616_m1), IFNγ (Mm00801778_m1), CXCL10 (Mm00445235_m1), CCL5 (Mm01302427_m1), TGFβ (Mm00441724_m1), Mx1 (Mm00487796_m1), Camp (Mm00438285_m1), IFNβ (Mm00439552_s1), IFNα (Mm03030145_gH) and SDHA (Mm01352366_m1). The following primers were used to quantify mRNA expression: mouse L12α (Forward (F), 5′GTACCAGACAGAGTTCCAGG; Reverse (R), 5′CGCAGAGTCTCGCCATTATG), IFNκ (F, 5′CTGACAGTCTACCTGGAGTTG; R, 5′GTTCTTGCTTGAAGGTGGGTG), iNOS (F, 5′CTCGGAGGTTCACCTCACTG; R, 5′GTGCTGCAGACACCATGGTG), IRF7 (F, 5′GAGCAAGACCGTGTTTACG; R, 5′CATGATGGTCACATCCAGG), and Raet1e (F, 5′AGCAGTGACCAAGCGCCATC; R, 5′CCTTGATGGTCAAGTTGCAC). Samples prepared without reverse transcription enzyme served as negative control templates. Values were normalized to SDHA expression because of its suitability as a reference gene in UVB-irradiated keratinocytes [59] and data were expressed as arbitrary units.

### Protein extracts, co-immunoprecipitation, pull-down and western blotting analyses

Whole cell and skin extracts for western blotting were obtained in ice-cold lysis buffer containing 1% Nonidet P-40, 1% deoxycholate, 0.1% SDS, 50 mmol/L HEPES pH 7.5, and 150 mmol/L NaCl, together with protease and phosphatase inhibitors (10 μg/ml aprotinin, 10 μg/ml leupeptin, 86 μg/ml iodoacetamide, and 1 mM PMSF, 1 mM Na_3_VO_4_) as described [6]. Histones were acid extracted with 0.2 M HCL. Cell extracts for co-immunoprecipitation and pull-down assays were obtained in ice-cold lysis buffer containing 1% NP-40, 10% glycerol, 50 mmol/L HEPES pH 7.5, 150 mmol/L NaCl, and the previously mentioned inhibitors. Immunoprecipitated complexes were washed four times with buffer containing 20 mmol/L HEPES pH 7.5, 0.1% Nonidet P-40, 10% glycerol, and 150 mmol/L NaCl. Samples were boiled in Laemmli buffer and separated by SDS-PAGE. Western blotting was performed as previously described (Parra et al, 2009). Primary antibodies used throughout this study include, rabbit anti-mouseTREX2 (1/1000; in house [6]), anti-phospho-STAT1 (Tyr701), anti-phospho-STAT3 (Tyr705) (1/1000; Cell Signaling), and anti-H2AX (1/1000; Abcam), and mouse anti-γH2AX (Ser139), anti-βactin (1/10000; Sigma), and anti-GFP (1/2000; Roche). Horseradish peroxidase-conjugated goat anti-rabbit or goat anti-mouse antibodies (1/10000; BioRad) were used as secondary antibodies. Detection was performed with enhanced chemiluminescence (ECL, Biological Industries). For co-immunoprecipitation, 1 mg of protein extracts from untreated and UVB-irradiated HACAT_YFP and HACAT_YFP-TREX2 cells were incubated with anti-GFP magnetic beads (MBL). For pull-down assays, 2 mg of protein extracts from untreated and UVB-irradiated keratinocytes were incubated with MBP or MBP-TREX2 fusion proteins bound to amylose magnetic beads (New England Biolabs).

### Statistical analyses

Statistical analyses were conducted using GraphPad Prism 5 Software™. The Mann-Whitney test was used for analysis of tumor multiplicity at single time points and for comparisons between mouse genotypes or treatment conditions. Tumor incidence was analyzed using the Log-rank (Mantel-Cox). The unpaired Student's *t* test was applied for analysis of keratinocyte data. Associations between TREX2 expression and categorical variables of SCCs were analyzed using the χ2 and Fisher's exact tests, as appropriate. *P* values of ≤ 0.05 were considered to be statistically significant.

## SUPPLEMENTARY FIGURES


