# Supplementary material for: Multifaceted role of TREX2 in the skin defense against UV-induced skin carcinogenesis
Source: Oncotarget. 2015 Jun 15;6(26):22375–96. doi: 10.18632/oncotarget.4296 (PMC4673170; doi:10.18632/oncotarget.4296)
Supplement: Supplementary file 1 [file oncotarget-06-22375-s001.pdf]

SUPPLEMENTARY FIGURES

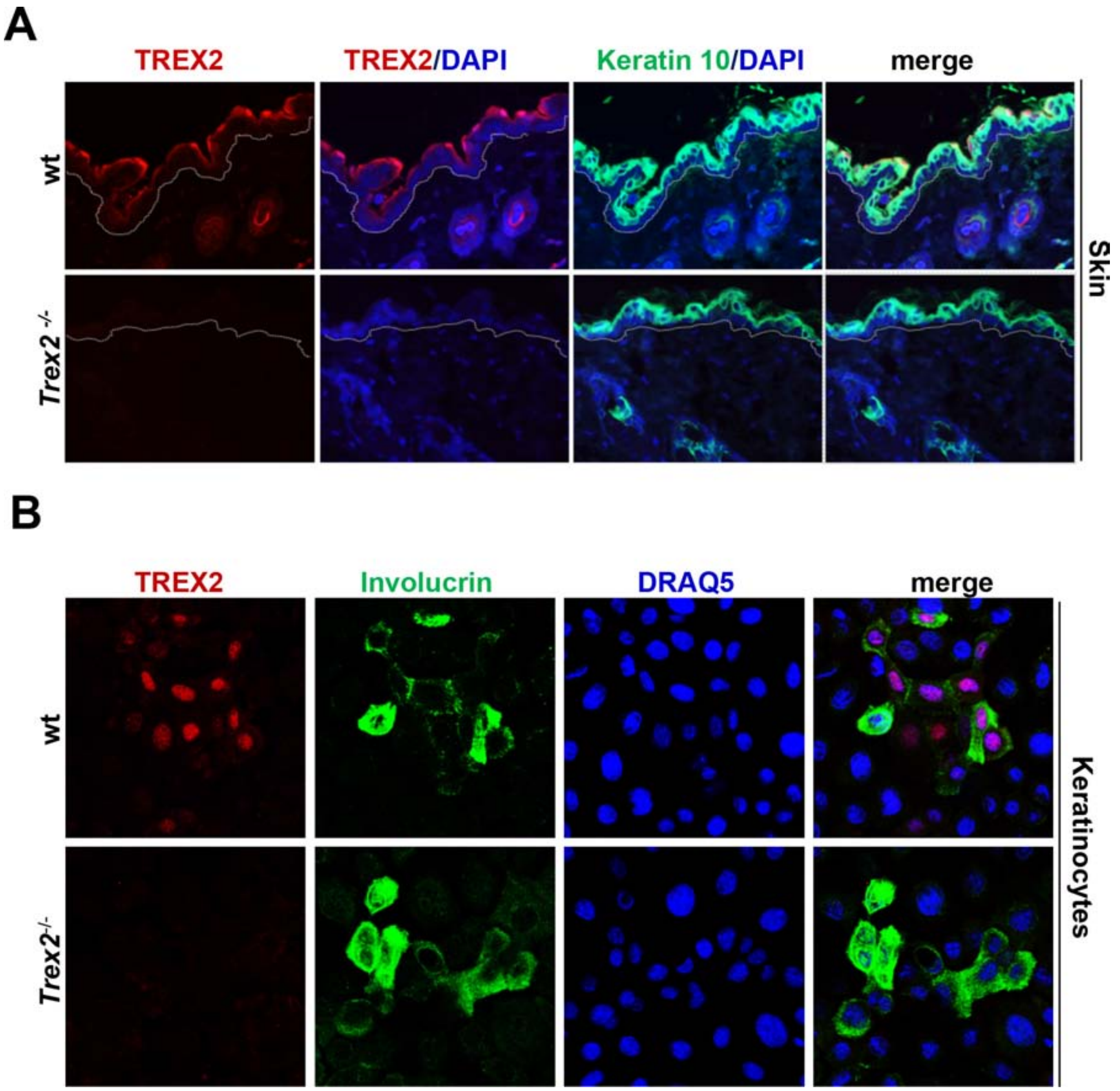

Supplementary Figure S1: *TREX2* expression in mouse skin and keratinocytes. A. Skin and **B.** primary keratinocytes from wt and *Trex2*$^{-/-}$mice were stained with antibodies against *TREX2*, keratin-10 and involucrin, as indicated. Nuclei were counterstained with DAPI or DRAQ5, as indicated. White dotted lines demarcate the epidermal-dermal border. Original magnification was 20x (A) and 63x (B).

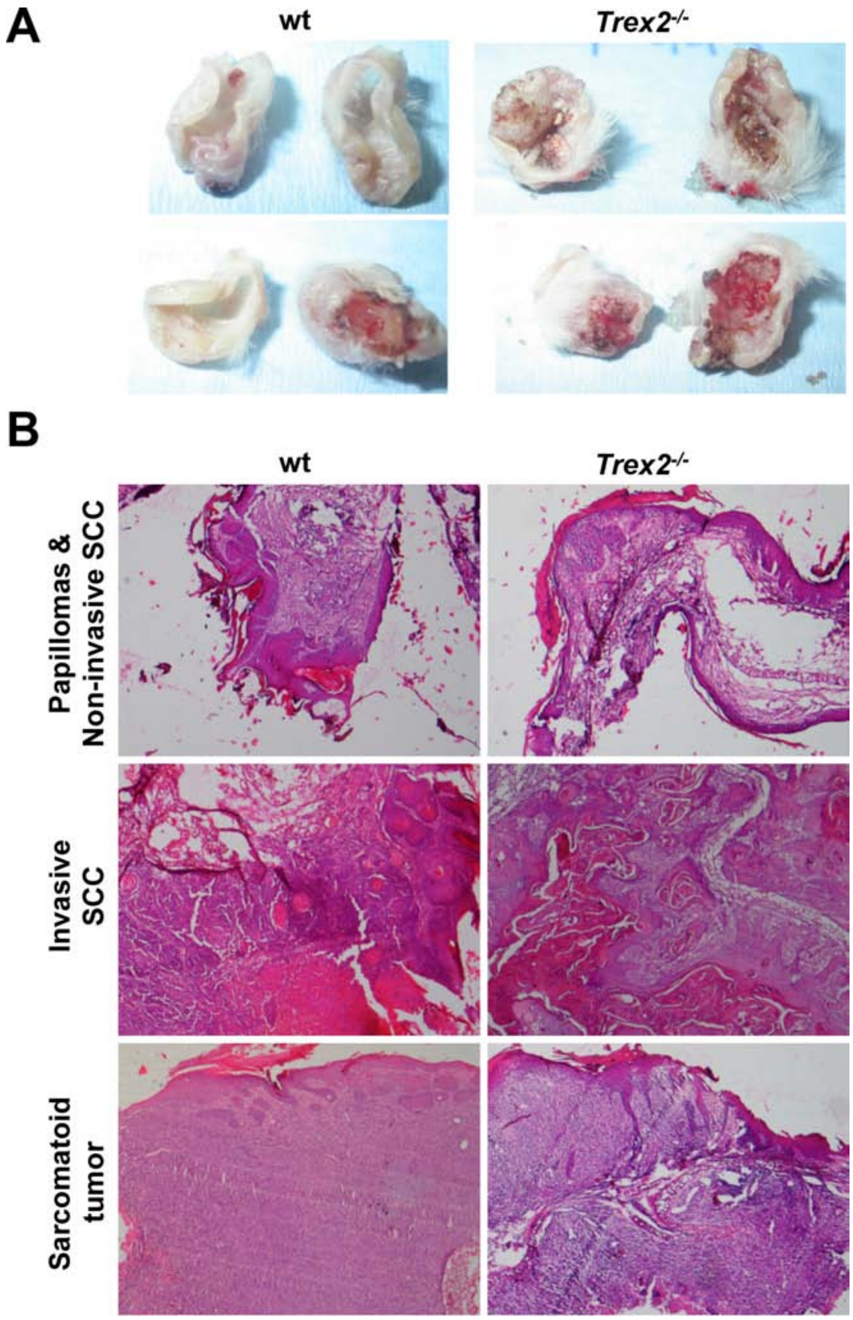

Supplementary Figure S2: UVB-induced skin carcinogenesis in *Trex2*$^{-/-}$ mice. A. Representative macroscopic images and **B.** histological sections from tumors in ears of wt and *Trex2*$^{-/-}$ mice. Original magnification was 4x.

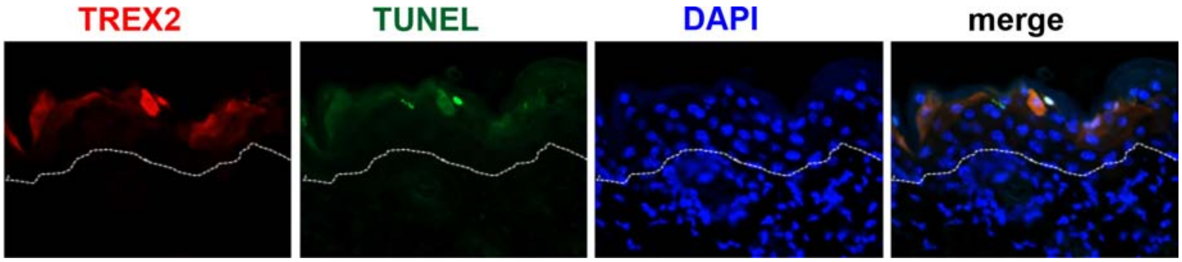

Supplementary Figure S3: *TREX2* and TUNEL staining in chronic UVB-irradiated skin of wt mice. Nuclei were counterstained with DAPI. White dotted lines demarcate the epidermal-dermal border. Representative images are shown. Original magnification, 40x.

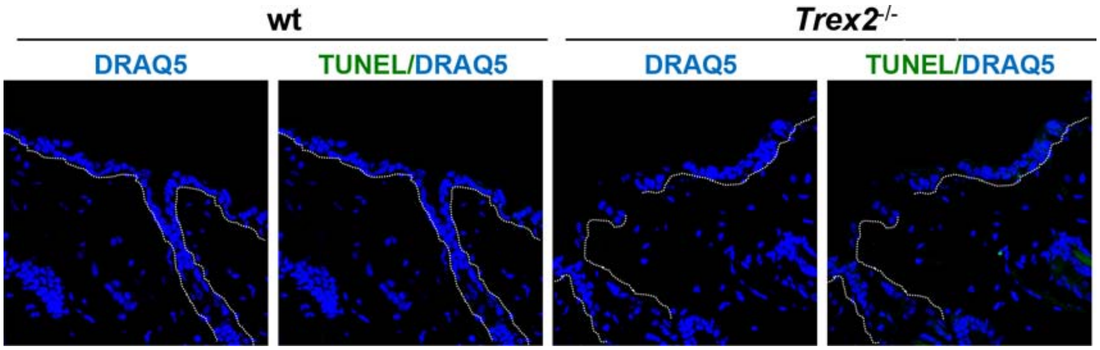

Supplementary Figure S4: Cell death in normal skin of wt and *Trex2*$^{-/-}$ mice. TUNEL and active caspase-3 staining in non-irradiated skin of wt and *Trex2*$^{-/-}$ mice. Nuclei were counterstained with DRAQ5. White dotted lines demarcate the epidermal-dermal border. Representative images are shown. Original magnification, 40x.

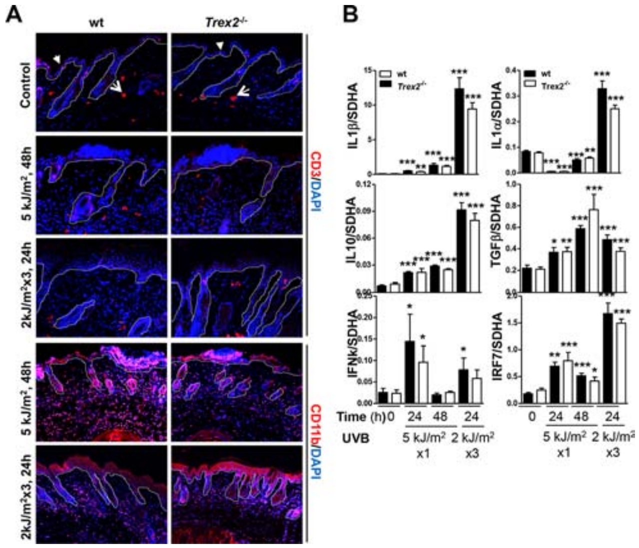

Supplementary Figure S5: UVB-induced inflammatory response in wt and *Trex2*$^{-/-}$ skin. UVB-induced inflammatory response in wt and *Trex2*$^{-/-}$skin. **A.** Immunodetection of CD11b and CD3 expression. Nuclei were counterstained with DAPI. Original magnification was 20x (CD3) and 10x (CD11b). White dotted lines demarcate the epidermal-dermal border. **B.** Expression of immune genes in acute and chronic UVB-treated skin of wt and *Trex2*$^{-/-}$ mice. The mRNA levels were determined by RT-qPCR. Graphs show mean values and SEM of at least five mice from each condition and genotype. Significant differences by Mann-Whitney test between controls and UVB-irradiated samples: *$P < 0.05$, **$P < 0.01$, ***$P < 0.001$. There are no significant differences between wt and *Trex2*$^{-/-}$ skin samples.

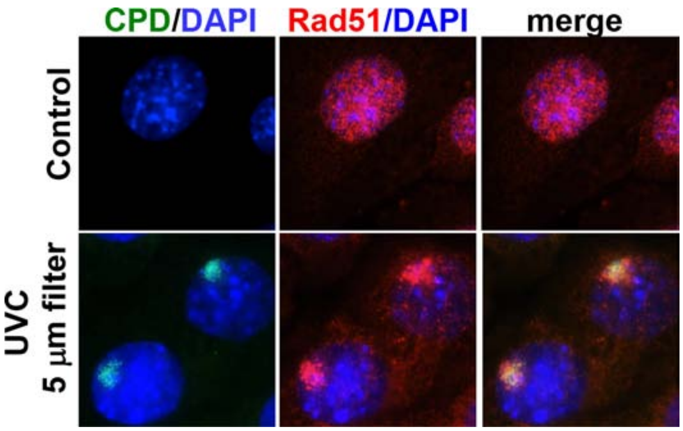

Supplementary Figure S6: Immunodetection of UVB-induced DNA damage. Immunodetection of UV-induced DNA damage. Rad51 recruitment to sites of localized UV DNA damage. Mouse keratinocytes that were untreated or treated with UVC (100 J/m^2) through a 5-mm micropore filter were immunostained with the indicated antibodies. Nuclei were counterstained with DAPI. Original magnification was 100x.
